# Work-Related Musculoskeletal Injury Rates, Risk Factors, and Ergonomics in Different Endoscopic Specialties: A Review

**DOI:** 10.3390/healthcare12090885

**Published:** 2024-04-24

**Authors:** Veronica Bessone, Daniel B. Roppenecker, Sven Adamsen

**Affiliations:** 1 Ambu Innovation GmbH, Karl-Drais-Strasse 4B, DE-86159 Augsburg, Germany; 2 Ambu A/S, Baltorpbakken 13, DK-2750 Ballerup, Denmark; 3 Digestive Disease Center, Bispebjerg Hospital, University of Copenhagen, Bispebjerg Bakke 23, DK-2400 Copenhagen, Denmark

**Keywords:** ergonomics, injury prevention, gastrointestinal endoscopy, endourology, bronchoscopy, nasal endoscopy

## Abstract

Endoscopy-related musculoskeletal injuries (ERIs) are frequent among gastrointestinal, pulmonary, nasal, and urologic endoscopists, impacting the healthcare system. The present review aims to compare the ERI rates, risk factors, and ergonomic recommendations in the different endoscopic fields. A review was conducted using PubMed and Cochrane Library for articles based on surveys and published until 10 January 2024. Demographic, work, and ERI data from 46 publications were included, covering 10,539 responders. The ERI incidence ranged between 14% and 97%, highlighting the need of intervention independent of the specialties. The neck, back, and shoulder were the most frequent ERI locations, while gender, age, years of experience, and procedure volume the most common risk factors. Ergonomic recommendations suggest concentrating on endoscope design changes, especially in gastrointestinal endoscopy, to increase the comfort, adaptability of the equipment in the operating room, and workflow/institutional policy changes. The inclusion of an ergonomic timeout guarantees the correct equipment positioning, the neutralisation of the endoscopist’s posture, and an indirect break between procedures. Ergonomic training to increase awareness and best practice should be promoted, also using new technologies. Future research should concentrate on intervention and comparative studies to evaluate to which extent prevention measures and newly designed equipment could reduce ERI incidence.

## 1. Introduction

Endoscopy requires the physical interaction between the clinician and the endoscope to insert, advance, and manoeuvre the tip. Independent of the field—gastrointestinal (GI) endoscopy (gastro-, duodeno-, and colonoscopy), bronchoscopy, rhinolaryngoscopy (ear–nose–throat (ENT) or nasal endoscopy), or endourology (uretero-, nephro-, and cystoscopy—the clinician operates in a constrained space, interacting with medical staff and equipment, and often in awkward positions. Endoscopists perform several procedures with limited breaks including movement repetitions and a high physical demand, resembling those of athletes [1,2]. Due to generally limited or no ergonomic awareness so far, endoscopy-related (musculoskeletal) injuries (ERIs) occur in all the mentioned specialties [3,4,5,6,7,8,9,10,11,12,13,14,15,16,17,18,19,20,21,22,23,24,25,26,27,28,29,30,31,32,33,34,35,36,37,38,39,40,41,42,43,44,45,46,47,48], with a related impact on the health care system and on the private and work life of the injured endoscopists [1,23,25].

Endoscopists spend most of their occupational time actively performing endoscopies. The weekly average is 16 h for urologists [48], 29 h for otorhinolaryngologists [39], and 25 h for GI endoscopists [15], who perform more than 20 GI procedures per week [18,21,24,25,29].

The time spent actively using the endoscope and the procedure volumes have been identified as risk factors for developing ERIs [3,10,15,20,25,37]. Other risk factors include operating in a non-ergonomic set-up [4] and using medical devices that are (usually) not ergonomically designed [49]. With an ERI incidence of up to 97% among otorhinolaryngologists [37], endoscopists need to modify their practice due to an ERI [4,6,7,8,10,12,13,14,15,16,22,25,27,31,32,35,36,38,39,43], or to be absent from work for up to 210 days [37]. Despite the ERI impacts on procedure performance and on the health care system [1,23,25], some fields of endoscopy, such as bronchoscopy, still lack studies in the field of ergonomics and prevention. A comparison of ERI impact, risk factors, and ergonomic recommendations between the different endoscopic specialties could be beneficial for increasing awareness. It could also help transfer preventive measures and recommendations from a more investigated specialty, such as GI endoscopy, to a less investigated one.

In GI endoscopy, the awareness of ERIs and the acknowledged need for better ergonomics are also demonstrated by the testimonials of active endoscopists [50,51]. To cite one example, a young female GI endoscopist reported how at the beginning of her fellowship, moved by enthusiasm and passion, she performed procedures without concentrating on body posture and on endoscope manoeuvring, but she soon developed pain in her shoulder and wrist [51]. Since the sub-optimally designed endoscope and her anthropometrics (small hands) were aspects that she could not modify, she concentrated on her body posture and the design of the operating room to reduce the pain and improve the ergonomics, with immediate positive effects on her health. The testimony concludes and illustrates the importance of focusing on ergonomics from the very beginning of endoscopic training. In addition, with endoscopy being highly repetitive, muscle memory developed while maintaining wrong postures in the early stages of a career may be hard to change.

With the expected increasing demand for diagnostic and therapeutic endoscopic procedures [52,53,54], an increased focus on ERI awareness and prevention measures is needed in all specialties to improve the work quality of the professionals and the efficiency of the health care system. Ergonomics can in fact guarantee productivity enhancement, error reduction, increased safety, and comfort maximisation, by applying principles to analyse and optimise the interaction between humans and tools, tasks, and environment in a working space [55].

To the best of our knowledge, no studies have been conducted to compare musculoskeletal ERIs and risk factors between GI and nasal endoscopy, bronchoscopy, and endourology. Therefore, the goal of this study is to perform a review to assess and compare the musculoskeletal ERI incidence and locations, the risk factors, and ergonomic recommendations among the clinical professionals (doctors, surgeons, or nurses) of these endoscopic specialties. Additionally, an overview of the suggested ergonomic preventive measures is given.

## 2. Materials and Methods

A review of peer-reviewed studies focusing on ergonomics and ERIs among active professionals in GI, nasal, pulmonary, and urologic endoscopy was conducted. The identification and screening process of the articles was based on the Preferred Reporting Items for Systematic Reviews and Meta-Analyses (PRISMA) guidelines [56]. 

### 2.1. Identification

The comprehensive literature search on musculoskeletal ERIs and ergonomics in the different endoscopic specialties was performed by means of PubMed and Cochrane Library. To retrieve the publications in English language, the keywords “musculoskeletal injuries” or “ergonomics” and “GI endoscopy”, “endoscopy”, “laryngoscopy”, “bronchoscopy”, “ENT”, “nasal endoscopy”, or “urology” were used, respectively. The reviewed papers were published until and including 10 January 2024.

During the identification phase, from the retrieved lists of papers, the ones presenting the same titles and authors were excluded, being double publications.

### 2.2. Screening

To limit and focus the search, the inclusion criterium of the publications for this review was a survey with endoscopy professionals as respondents. Since the current study concentrated on musculoskeletal ERIs, only articles on work-related injuries and pain affecting muscles or joints were included. Therefore, studies focusing exclusively on mental diseases (such as breakdown, depression, stress), dermatological issues (such as chemical burns or cuts), or ophthalmological diseases (such as cataract) were excluded. Studies reporting ERIs and mental, dermatological, or ophthalmological diseases were included, but only data regarding musculoskeletal injuries were extracted and reported in the results.

At first, the titles and then the abstracts were screened. In this phase, criteria of exclusion were non-fitting content and/or abstract missing. Afterwards, the papers were screened using the additional exclusion criteria of being not in English or being a secondary publication (such as a review or a letter to the editor). Finally, the full texts of the remaining papers were read. Any remaining papers with topic not fitting the purpose of this review were excluded.

To broaden the results, the excluded review articles reporting studies based on surveys focused on ERI incidence in endoscopy were also screened. The reported publications were analysed with the criteria of exclusion, compared with the list of retrieved publications, and eventually added to the list of reviewed publications when missing.

To facilitate the screening process, we utilised Microsoft Excel (Microsoft 365, Version 2302, Microsoft Corporation, Redmond, WA, USA). Specifically, we applied the filter function to exclude papers containing “laparoscopic” or “robotic” in the title.

### 2.3. Data Analysis

The results are reported and discussed according to the PECO format (population, exposure, comparator, and outcomes) [57] (Table 1). The population characteristics are reported in (Section 3.2) with demographic, anthropometric and work data, while (Section 3.3) reports ERI impact (incidence, location, type, leave, treatment, modifications to the practice) in the different endoscopic specialties. The exposure, here considered as the identified ERI risk factors, is displayed in (Section 3.4) and shows only data reported to be statistically significant (*p* < 0.05) in the original articles. Finally, (Section 3.5) is an overview of the outcomes as suggested ergonomic preventive measures in the different fields of endoscopy.

For simplicity, the data are reported in percentages relative to the number of responders. Exceptions are clearly stated when present as well if the data were calculated, merged, or rounded for clarity.

## 3. Results

### 3.1. Characteristics of the Reviewed Studies

**Figure 1:**
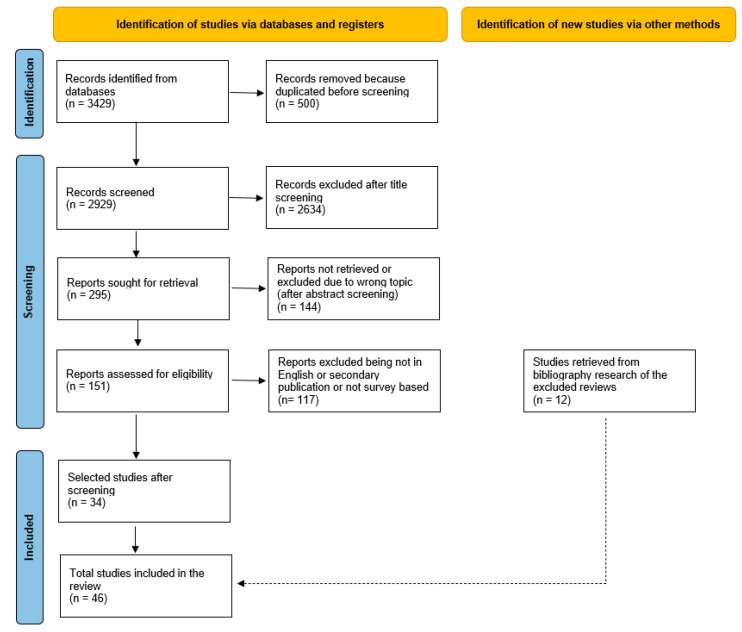
Literature research flow chart.

A total of 3429 articles were retrieved by means of the electronic database search. After reviewing titles, abstracts, and texts according to the exclusion criteria, 34 publications were identified to be included (Figure 1). From the excluded review articles, 12 additional publications reported in those reviews and dealing with ERI incidence in endoscopy were added to the list. Therefore, a total of 46 papers were included in the present review and their aims reported in Appendix A (Table A1). Despite the reviewed studies being all based on surveys, the number of questions, type, and formulation were not always possible to retrieve, resulting in possible missing data.

**Figure 2:**
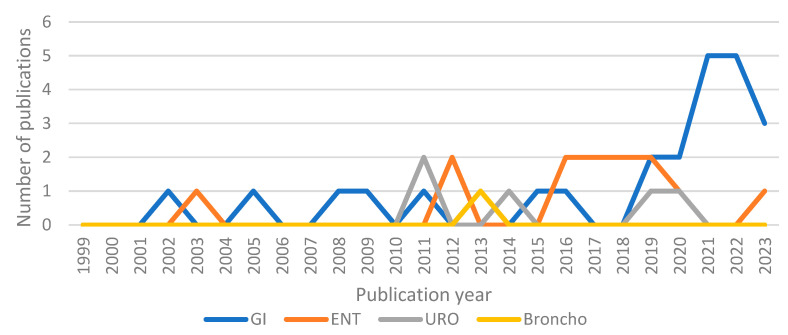
Number of included publications in relation to the publication year (GI: gastrointestinal endoscopy; ENT: nasal endoscopy/rhinolaryngoscopy; URO: endourology; Broncho: bronchoscopy). Until the review date (10 January 2024), only one paper for GI endoscopy was published in 2024 and not included in the graphic to avoid misinterpretation.

Twenty-seven articles were within GI endoscopy [3,4,5,6,7,8,9,10,11,12,13,14,15,16,17,18,19,20,21,22,23,24,25,26,27,28,29], with thirteen within nasal endoscopy [30,31,32,33,34,35,36,37,38,39,40,41,42], one within bronchoscopy [43], and five within endourology [44,45,46,47,48]. Of the 46 articles, 34 were published between 2013 and 2023, and 25 between 2018 and 2023, demonstrating an increased interest in ERIs and ergonomics, especially in GI endoscopy, where around 70% of the reviewed papers were published after 2018 (Figure 2). Some studies focused on one type of endoscopy only, for instance, colonoscopy alone for GI endoscopy, while others covered all procedures within a specialty.

The retrieved studies were based on surveys shared nationally (for instance, in the United States of America, Pakistan, Canada, the United Kingdom) and internationally (Table A1). The surveys were shared online through societies’ mailing lists and/or distributed as printed versions during scientific conferences. The number and type of questions as well as the type of answers (close/open, options) were not always possible to retrieve. The number of questions in the survey ranged between 7 [26,44], and 56 [28]. The highest number of recipients was 15,868 [20,23], and the study with the highest number of responders included 1698 subjects [20].

**Figure 3:**
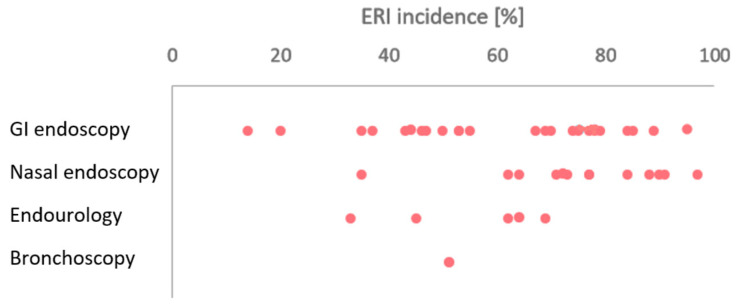
Distribution of the endoscopy-related injury (ERI) incidence (in %) in the different specialties reported in the reviewed articles (GI: gastrointestinal).

Since the studies differed from each other with respect to methodologies and data analysis and report, a meta-analysis was not possible. Therefore, the results were organised and summarised in tables (Table 2, Table 3, Table 4, Table A2 and Table A3) and figure (Figure 3) and the data discussed in a narrative way.

### 3.2. Demographic, Anthropometric, and Work Data

In this section, demographic, anthropometric, and work data of the population (P) are reported (Table A2, Appendix A). Cumulated data from 10,539 healthcare professionals (endoscopists, surgeons, and nurses) of the four different specialties of endoscopy were included: 6589 GI endoscopists, 2353 otorhinolaryngologists, 1437 urologists, and 160 bronchoscopists. Due to anonymity, the participation of responders in more than one survey could not be excluded. The numbers of responders of the retrieved studies varied from 45 [41] to 1698 [20].

The ratio of males varied from 45% [15,27] to 98% [14], with the number of female responders being higher in only two studies [15,27]. The age and years of experience in endoscopy varied among the studies, with some concentrating exclusively on fellows and having over 80% of the responders as being younger than 35 years [11,12,13]. When reported, the responders described to be active in terms of regularly performing endoscopy in most of the studies [4,6,14,19,22,25,27,29,31,33,35,41].

Depending on the focus of the surveys, the volume of endoscopic procedures was reported differently. For instance, specifying the type of procedure, its frequency, or the active use of endoscopes varied (Table A2). Additionally, the volume and/or active hours of endoscopic work depended on the endoscopic specialties, or the type of procedure.

### 3.3. ERI Impact

This section summarises the ERI incidence, location, type, and treatment of the population (P). A detailed overview can be found in Table A3 (Appendix A). It is important to mention that some publications make the distinction between injury and pain, others consider pain as a general disease, and other studies do not make any distinction.

The weighted ERI incidence average reported in the studies was 60% in GI endoscopy, 76% in nasal endoscopy, 51% in endourology, and 51% in bronchoscopy and displayed in Figure 3, while Table 2 provides a summary of the main ERI impact results.

**Table 2:** Summary of the endoscopic-related injuries (ERIs) impact in the different fields of endoscopy.

Specialty and Number of Publications	Gastrointestinal Endoscopy (27)	Nasal Endoscopy (13)	Bronchoscopy (1)	Endourology (5)
Overall ERI incidence	14–95%	35–97%	51%	33–69%
Female ERI incidence	13–84%	87–93%	61%	33–39%
Male ERI incidence	12–87%	58–87%	49%	46–70%
Most common ERI locations	Neck, back, and hand/thumb	Neck	Neck and back	Neck and back
Most common ERI types	Pain	Pain, neck stiffness	Pain	Pain
Leave rate	1–30%	6–23%	6%	8%
Most common treatments	Physiotherapy, medication	Physiotherapy	Medication	Medication, physiotherapy
Main modifications practice due to ERIs	Stretching, reduce number of procedures, more breaks	Reduce of number of procedures	Modify workspace, reduce number of procedures	-

In most of the studies on GI and nasal endoscopies, the ERI rate was higher among the female participants (up to 93% [39]). In endourology, the ERI incidence was higher among males [47,48].

The neck, back, and shoulder were the most frequent ERI locations for all specialties. The majority of endourologists, bronchoscopists, and otorhinolaryngologists reported the neck being the most frequent ERI location, with rates of up to 82% [36,38]. In GI endoscopy, high incidences were reported for the thumb (up to 63% [20]), hand (79% [28]) and wrist (82% [11]). Besides unspecified “pain”, numbness, De Quervain tenosynovitis, and carpal tunnel syndrome were the most frequently reported ERI types. Some articles specified that endoscopists reported simultaneous ERIs in different locations [6,20,43,44].

The injured endoscopists underwent different treatments, including physiotherapy and massages, medications, rest, and the use of splints/braces. Between 10% [19] and 60% [9] of the injured endoscopists sought medical care, with up to 29% requiring corrective surgery [31], often for treating carpal tunnel syndrome [20,23]. Due to an inability to perform due to pain or surgery, up to 30% had to take leave from work due to ERIs [21], with time off of up to 210 days in one study [37]. Besides affecting professional life, endoscopists also reported ERI symptoms occurring during daily life, as highlighted by [15], in which 45% of responders mentioned that the symptoms were present while performing endoscopy, as well as in private life.

Between 12% and 69% reported modifications to their practice due to ERIs. These included technique modifications, reduction in the procedure volume, increased number and duration of breaks, stretching and rest, use of orthopaedic and/or supporting devices, and use of adjustable equipment, such as a monitor, table, or chair [4,6,7,8,10,12,13,14,15,16,22,25,27,31,32,35,36,38,39,43].

In GI endoscopy, pregnancy worsened (70% [20]) or resulted in new ERIs (79% [20]) and led to procedure modifications (as reduction in procedure volume or performing while sitting [29]), while no information was reported for the other specialties. Pregnancy did not stop gastroenterologists carrying out procedures, and 23% also continued performing procedures requiring fluoroscopy [20].

### 3.4. Risk Factors

In this section, the risk factors considered as exposure (E) are reported. The risk factors reported to statistically correlate with ERI incidence are displayed in Table 3. It is relevant to mention that the presumed ERI causes according to the endoscopists reflected the risk factors highlighted by statistics. In particular, endoscopists consider as main presumed ERI causes the application of torque with the right hand [5,7,16,20,23,27], standing in awkward positions [16,20,23,27,35] for prolonged periods of time [5,7,16,20,23,27,35], repetitive movements [16,35], the manipulation of the knobs with the left hand [5,7,16,20,23,27], patient positioning [27], the use of a lead apron [4,20] and of the elevator in duodenoscopes [20], a lack of breaks [4], and non-adjustable monitor/table set-up [4,5,16,20,23,35].

Female professionals resulted in having a higher risk of developing ERIs among GI [12,15,16,20,21,23,27,29] and ENT endoscopists [39], but gender was not found to be an ERI risk factor in bronchoscopy (one survey only) and endourology. On the other hand, z correlation between smaller hand size and ERI incidence was found in bronchoscopy [43].

Age is highlighted to be a risk factor, but, depending on the study and specialty, both younger and older endoscopists were statistically more affected by ERIs. Higher age [8,9,20,25], and longer experience in endoscopy [8,9,15,20,23] were reported to be risk factors in GI endoscopy. In bronchoscopy and endourology, a higher rate of ERIs was found among younger professionals, which can be caused by the general poor ergonomics of younger endoscopists demonstrated by higher Rapid Upper Limb Assessment (RULA) scores [58]. Finally, in nasal endoscopy and endourology, higher age and longer working experience were risk factors for developing ERIs in the wrist, elbow, shoulder [37], hand, neck, and back [44], respectively. On the other hand, younger bronchoscopists and urologists were more frequently affected by ERIs than older ones [43,46].

In GI endoscopy, higher endoscopist weight [10,21], height [15], and BMI [19] correlated with higher ERI incidence. Higher weight and height are risk factors in developing ERIs located in the hip, knee, and ankle [21].

Higher procedural volume and duration correlated with higher ERI incidence in GI endoscopy [3,5,10,15,17,18,20,22,25] and in ENT [33,37,41], but not in endourology and bronchoscopy. Specific to the procedure, only in endourology were some risk factors identified as being significant: endoscopists who perform a transurethral resection of the prostate [46] or where the patient’s prostate volume is greater than 75 g [48] had higher ERI incidence than that of other endourologists.

**Table 3:** Risk factors statistically (*p* < 0.05) influencing the rate of endoscopy-related injuries (ERIs) in the different specialties (CTS: carpal tunnel syndrome; DQT: De Quervain tenosynovitis; ERCP: endoscopic retrograde cholangiopancreatography; EGD: esophagogastroduodenoscopy; ESD: endoscopic submucosal dissection).

				Gastrointestinal Endoscopy	Nasal Endoscopy	Bronchoscopy	Endourology
**Demographic**	**Gender**	**Among female**	Higher ERI rate	[12,15,16,20,21,23,27,29]	[39]		
			Higher [location] ERI rate	Left thumb: [20,23] Fingers: [20,27] Hand: [27] Wrist: [20,29] Shoulder: [27,28] Neck: [28] Upper back: [20] Foot: [22]	Wrist: [36] Hand: [36] Neck: [36]		
			Higher [ERI type] rate	Hand/arm numbness: [20] CTS: [20] DQT: [20]			
			Higher time off likeness	[16]			
		**Among male**	Higher [location] ERI rate	Elbow: [20,23] Lower back: [20]			
			Higher rate of leave connected to number of ERIs	[20]			
	**Age**	**Higher age**	Higher ERI rate	[8,9,20,25]			
			Higher [location] ERI rate				Hand: [44] Neck: [44] Back: [44]
			Higher ERI severity	[22]			
		**Lower age**	Higher ERI rate			[43]	[46]
	**Others**	**Higher body mass index**	Higher ERI rate	[19]			
		**Higher weight**	Higher ERI rate	[10]			
			Higher [location] ERI rate	Hip: [21] Knee: [21] Ankle: [21]			
		**Higher height**	Higher ERI rate	[15]			
			Higher [location] ERI rate	Knee: [21] Ankle: [21]			
		**Smaller glove size**	Higher ERI rate			[43]	
**Work characteristics**	**Years of experience**	**Longer experience**	Higher ERI rate	[9,10,15,20,23]			
			Higher [location] ERI rate		Right wrist: [37] Right elbow: [37] Right shoulder: [37]		Neck: [44] Back: [44]
		**Lower experience**	Higher ERI rate			[43]	
		**Longer experience in ERCP**	Higher ERI rate	[4]			
	**Endoscopic volume**	**Higher procedural rate**	Higher ERI rate	[3,10,18]			
			Higher [location] ERI rate	Hand: [3] Thumb: [3] Elbow: [3] Lower back: [3]			
		**Higher number of [frequency] procedures**	Higher ERI rate	Weekly: [3,10,20]			
			Higher [location] ERI rate		Yearly; hip/thigh: [33]		
		**More [frequency]**	Higher ERI rate	Colonoscopies/week: [5,15,20]	16 h/week: [37]		
				EGD/week: [10,15]	8 h/day: [41]		
				ERCP/year: [17]			
				Endoscopy time/week: [3,15,25]			
			Higher ERI severity	Sigmoidoscopy/ileoscopy/pouchoscopy/year: [22]			
	**Others**		Higher ERI rate	Increased procedural duration: [18] General practice: [20]			
			Higher shoulder ERI rate	Higher EGD volume: [22] Lower ESD (1–90 min/month): [19]			
			Higher lower back ERI rate	Longer upper ESD (≥181 min/month): [19] Lower gastrointestinal treatment (≥526 min/month): [19] Lower ESD (1–90 min/month): [19]			
			Higher hand/wrist ERI rate	Lower yearly colonoscopies: [22] Longer colonoscopy insertion time-related: [22] Lower EGD volume: [22]			
**Others**		Higher ERI rate among endoscopists who […]		… lack ergonomic training: [13] … lack breaks between procedures: [18,20] …carry out the procedure while standing: [6] (during upper gastrointestinal)	… operate with beam splitter or endoscope alone: [33] … operate at the right of the table: [33] … are right-handed: [33] … carry out the procedure while standing has higher neck ERI rate: [40] …carry out the procedure while standing: [33] (during endoscopy sinus surgery)		… performs benign prostatic hyperplasia with transurethral resection of the prostate: [46] … performs benign prostatic hyperplasia, where the patient prostate volume is >75 g: [48]

Performing the procedure while standing was reported to be a risk factor in GI [6] and nasal endoscopy [33], two specialties in which performing while sitting is more infrequent than in endourology (in GI endoscopy sitting was reported between 4% [7] and 58% (in colonoscopy) [6], in nasal endoscopy up to 42% [37], and up to 76% in endourology [48]).

In general, the potential benefit of increasing awareness about ergonomics and ergonomic training was reflected in only one study, that found that the ERI rate was higher among GI endoscopists who did not undergo ergonomic training [13].

### 3.5. Recommendations for ERI Prevention

The outcomes considered as recommendations to improve ergonomics and reduce the ERI rate are reported in Table 4 and can be categorised in three groups: endoscope design, environment (set-up and material), and ergonomic best practice (procedure volume, schedule, and technique).

The first measure is prevention through design, meaning that the industry should develop human-factors-engineered endoscopes. The suggested endoscope design changes for GI endoscopy regard the handle size, weight, shape, and manipulators’ (lever and knobs) force/torque reduction [5,7,8,14,16,20,23,28,29]. Female gastroenterologists expressed the wish of increasing the availability of dial/knob extenders, and an optimisation of the endoscope size [28]. A total of 54% of GI endoscopists are willing to train on newly designed endoscopes [29]. In endourology, a reduction in the size of the endoscope and the construction of intuitive tip movement mechanics is proposed. Unspecified changes to the endoscope design are recommended in nasal endoscopy, bronchoscopy, and GI endoscopy [22,25,27,33,43].

An adequate maintenance of the equipment is recommended since with time the mechanical characteristics of the reusable endoscope changes [39]. As a result, the force needed to deflect the tip increases, making an already sub-optimally designed device even less ergonomic.

The adaptability of the equipment allows for the creation of an ergonomic space [4,7,16,25,31,32,35,39,40,43] and consequently accommodates all the user’s body structures. To do so, the room should be large enough to place all the equipment, such as the C-arm fluoroscope, laser lithotripsy machine, towers, ultrasound equipment, monitors, beds, and chairs. Additionally, all the necessary equipment and tools should be placed to be within easy reach [35]. The monitor, bed, table, and chairs should be adjustable to reduce strain on the neck, back, shoulder, and elbow [4,5,8,13,16,18,20,21,27,28,30,32,33,34,35,39,46,48]. In GI endoscopy [2], the recommendation is to have the table’s height in a neutral position and adjusted between 85 and 120 cm, depending on user height. The monitor should be placed directly in front of the endoscopists, at 52–182 cm to avoid neck rotation and 15–25° off from the horizontal line of the eye gaze. These recommendations are based on laparoscopic studies but can easily be transferred to other specialties. The use of a well-fitting [28], two-piece [4,27], and light [44] lead apron during fluoroscopy is recommended to reduce back pain. Promoting the performance of the procedure while sitting is a measure suggested for GI [7,21] and nasal endoscopy [30,40] to reduce neck, back, and foot pain. When the abovementioned recommendation is not possible, the use of a cushioned anti-fatigue mat is recommendable [2,7,8,20,27,28]. The foot pedals should be positioned to be within easy reach and fixed so that they are not moved during activation [39].

Ensuring an ergonomic posture, i.e., maintaining a neutral neck and back position, with body weight distributed evenly on the feet that are positioned apart, and avoiding hyperextension of the shoulder, back, and knee, is suggested to help decrease strain in the joints [18,27,29,34,35,39,41,46]. Except for GI endoscopes that are designed so that they are not possible to hold with the right hand, endoscopes should be held with the dominant hand while the other hand is manoeuvring the insertion portion. To maintain an ergonomic posture throughout the entire procedure, the supervision of an ergonomic-trained nurse could be beneficial, by verbally advising the endoscopist when the position is not ergonomic [12,13,48]. Additionally, the support of a nurse or fellow is indicated as a measure of ERI prevention by assisting the procedure and applying torque on the insertion portion [8,16,29,35,39].

A reduction in procedure volume has been recommended in GI [13,16,18,19,20,25,27] and nasal endoscopy [31,39]. Microbreaks (also called intraoperative breaks) should be implemented in all endoscopic specialties [20,27,34,35]. These are breaks of a few seconds, during which the endoscopists can rest the endoscope, shake their hands, and perform some stretching, which are helpful for increasing mental focus, enhancing performance, and reducing the endoscopist’s pain. Additionally, the use of robotic-assisted procedures has been suggested in endourology [46], while in GI endoscopy electrically driven endoscopes or an electric-powered wheel have been suggested to reduce the physical demand from the user side while operating [26,29].

Planning an ergonomic time-out between procedures is a suggested preventive measure that can be implemented in all specialties [13,18,19,20,21,24,27,28,35]. It consists of a time before a procedure during which the endoscopist and the staff ensure the adaptation of the height and position of the equipment, the neutralisation of the body posture, and a muscular warm up (stretching and mobility exercises) while indirectly ensuring a physical (and mental) break between procedures.

Regular physical activity is recommended for all specialties, for both prevention and intervention, to strengthen postural muscles, to increase the endurance that is necessary for long procedures [1,21,47], and to maintain the bodyweight under control with a consequent reduction in the load on the back and joint [47].

Between 7% [11] and 97% [18] of GI endoscopists, 24% [40] and 66% [31] of nasal endoscopists, and 5% of urologists [46] are trained in ergonomics, while no information could be retrieved for bronchoscopy. The promotion of ergonomic training among endoscopists, including the abovementioned best-practice recommendations, is suggested in all the specialties to reduce ERIs and increase awareness among professionals [8,10,11,12,13,14,15,17,18,21,22,23,24,25,26,27,28,29,30,31,32,33,36,37,39,41,42,44].

**Table 4:** Recommendations to reduce endoscopy-related injuries in the different specialties of endoscopy according to endoscope design, environment and set-up, and ergonomic best practice.

		Gastrointestinal Endoscopy	Nasal Endoscopy	Bronchoscopy	Endourology
**Endoscope design**	Handle size reduction	[7,8,16,20,23,28,29]			[46]
	Manipulator force reduction	[5,8,20,23]			
	Grip	[20]			
	Dial extenders	[5,28]			
	Shape	[14]			
	Weight reduction	[8]			
	Unspecified	[22,25,27]	[33]	[43]	
	Others	Rotatable connection of the umbilical cord to the endoscope processor: [29] Electric-powered wheel: [29]			Intuitive tip movement (up is up, down is down): [45]
**Environment**	Adjustable monitors	[5,8,13,16,18,20,21,27,28]	[30,32,33,35,39]		[46,48]
	Adjustable bed	[4,5,8,13,16,18,20,21,27]	[30,32,35,39]		[46,48]
	Adjustable chair (with back rest)		[30,32,34,35,39]		
	Lead apron	Well fitting: [28] Two-piece: [4,27]			Light: [44]
	Anti-fatigue matt	[7,8,20,27,28]			
	Operating room space and design	[4,7,16,25]	[31,32,35,39,40]	[43]	
	Others	Accessory design: [14] Use of wireless medical device: [18] Use of paediatric endoscope: [22,28] Use of videoscope: [4]	Endoscope holder: [35] Microscope position: [35] Use instruments in reach: [35] Organised trolley: [35] Correct use of pedals: [39] Proper instrument maintenance: [39]		
**Ergonomic best practice**	Promotion of ergonomic training	[8,10,11,12,13,14,15,17,18,21,22,23,24,25,26,27,28,29]	[30,31,32,33,36,37,39,41,42]		[44]
	Increase in physical activity	[4,21,23]	[33,35,39]		[47,48]
	Ergonomic timeout	[13,18,19,20,21,24,27,28]	[35]		
	Posture	[18,27,29]	[34,35,39,41]		[46]
	Endoscope technique	Neutral grip: [19,27] C position in colonoscopy: [27] Pinkie manoeuvre: [19] Reducing torque with right hand: [7]			
	Procedure technique	Eliminate manual handling activities, use of magnetic imaging, use of abdominal compression devices: [26]	[34]		Use of robotics: [46]
	Scheduled breaks	[4,12]	[39,41]		
	Microbreaks	[20,27]	[34,35]		
	Procedure schedule (reduction, hours)	[13,16,18,19,20,25,27]	[31,39]		
	Sitting	[7,21]	[30,40]		
	Support of assistant/fellow	[16,29] … to inform about wrong technique/posture: [12,13] …to apply torque on the insertion portion: [8]	[35,39]		… to inform about wrong technique/posture: [48]
	Others	Ergonomic assessment: [12] Use of orthopaedic shoes: [7] Warm up: [21] Reduce age of participating endoscopists: [25]	Double glove: [34]		Low bodyweight: [47]

## 4. Discussion

The findings of this review are compared (C) in this section and highlight the relevant ERI incidence rate in all specialties, caused mainly by procedure volume, gender, and age, but also indirectly by non-ergonomic endoscopic rooms and equipment. The main recommendations to reduce ERIs included redesigning equipment, a reduction in procedure volume, longer and more frequent breaks, the use of adjustable equipment, and the promotion of ergonomic training.

This review shows that there is a discrepancy between awareness with respect to ergonomics and ERIs in GI endoscopy compared to other fields, highlighted by the higher number of articles mapping ERI incidence published in this field compared to in the others (27 papers in GI versus 1 in pulmonary endoscopy) (Table 2). The higher number of publications in GI endoscopy might be explained by the common belief that the bigger and not anthropometrically designed GI endoscopes may lead to bad ergonomics, while in endourology, as well as nasal and pulmonary endoscopy, the endoscopes are smaller and lighter and can be manoeuvred by only one hand.

### 4.1. ERI Impact Comparison

The current study demonstrated a high ERI incidence in all the specialties, emphasizing the need for bringing attention to preventive measures. High ERI rates were found especially among GI endoscopists (60% on average) and otorhinolaryngologists (76%). The higher rate in GI endoscopy can be explained by the high forces and torques, long standing time, heavier endoscopes, and longer procedure time compared to those of the other specialties [1,2], while in nasal endoscopy, this can be explained by the non-ergonomic position held by the otorhinolaryngologists [40]. Despite the ERI incidence varying within specialities, the rates are worth attention: in 36 studies out of 45 (1 study is not considered since it did not report the ERI incidence), the ERI incidence was higher or equal to 50%. Through the years, the trend of the ERI rate did not decrease, signalling that despite the ergonomic recommendations of endoscopy societies and of the scientific community in general [59,60,61], there is still much to be done.

When reported, the ERI incidence was higher among female professionals in most of the studies in GI endoscopy and otorhinolaryngology (Table 2). On the contrary, in endourology the ERI incidence was higher for males than females (46% vs. 39% [46] and 70% vs. 33% [48]), most probably due to the small sample of female responders. In GI endoscopy, the higher rate among female professionals has been explained by the biologically lower force that a female can generate compared to a male [62], in the specific example of GI endoscopy, to rotate the knobs and levers, for instance [21]. In addition, since women generally have smaller hands, they have a higher probability of being in need to adapt their position and movements to the endoscope [20,51], decreasing comfort and movement efficiency, and probably increasing ERI risk, as occurring in bronchoscopy [43].

In all specialties, the neck, back, and shoulder were the most frequent ERI locations, with a rate of up to 82% for the neck [36,38]. A position maintained during the procedure, especially triggered by the position of the monitor, can be considered the main risk factor [6]. In GI endoscopy, high incidences were reported for the thumb (up to 63% [20]), hand (79% [28]), and wrist (82% [11]), caused by procedure- and endoscope-related manoeuvres, repetitive movements, and endoscope size differences compared to those in other fields of endoscopy [43]. Besides the unspecified “pain”, numbness, De Quervain tenosynovitis, and carpal tunnel syndrome were the most frequently reported ERI types. The causes of the latter two diseases have been explained by repetitive thumb movements and high torque applied by the wrist, common characteristics of all endoscopic fields by manipulating levers and knobs and manoeuvring the insertion tube [5,59].

### 4.2. Risk Factor Comparison

Age is highlighted as a risk factor, but, depending on the study and specialty, both younger and older endoscopists were statistically more frequently affected by ERIs. Higher age [8,9,20,25], and longer experience in endoscopy [8,9,15,20,23] were reported to be a risk factor in GI endoscopy. One explanation could be that due to longer durations and physical demand, GI endoscopy could affect more older physicians. Additionally, longer, more complex procedures may be performed by more experienced endoscopists rather than younger ones. Conversely, it has been reported that young GI endoscopy fellows experienced ERIs especially in the first year of practice, especially if they did not attend ergonomic training [13]. In nasal endoscopy and endourology, higher age and longer working experience were risk factors for developing ERIs in the wrist, elbow, shoulder [37], hand, neck, and back [44], respectively. On the other hand, younger bronchoscopists and urologists were more frequently affected by ERIs than older ones [43,46], probably because they might actively use the endoscope more than the experienced or senior endoscopists, who are normally teaching and mentoring them without performing [43]. In some cases, authors explained the higher ERI rates among younger endoscopists by a kind of “natural selection”, in which healthier endoscopists and the ones who could adapt in the most ergonomic way could continue performing though their careers compared with the ones who had early ERIs [43].

Higher procedural volume and duration correlated with higher ERI incidence in GI endoscopy [3,5,10,15,17,18,20,22,25] and in ENT [33,37,41], but not in endourology and bronchoscopy. However, it could be speculated that a reduction in the procedure volume and duration could also be beneficial in endourology and bronchoscopy. Specific to the procedure, some risk factors were identified as being significant only in endourology. Endoscopists, who perform transurethral resection of the prostate [46] or where the patient’s prostate volume is greater than 75 g [48], had higher ERI incidence, probably due to the longer procedure times required.

Performing the procedure while standing has been reported to be a risk factor in GI [6] and nasal endoscopy [33], two specialties in which performing while sitting is more infrequent than in endourology (in GI endoscopy, respectively, between 4% [7] and 58% (in colonoscopy) [6]; in nasal endoscopy up to 42% [37]; and up to 76% in endourology [48]). When standing, increased load is distributed on the back and, in addition, when pedals are used in the procedure, the endoscopist needs to frequently maintain one foot flexed on a pedal while loading all body weight on the other foot.

### 4.3. Recommendations

The recommendations stated in the reviewed articles and summarised in Table 4 can be categorised under three aspects: endoscope design improvement, equipment and operating room design, and workflow/institutional policy changes.

#### 4.3.1. Endoscope Design Improvement

The first measure is prevention through design, by means of development by endoscope companies of human-factors-engineered endoscopes. Except for GI endoscopes that are designed so that it is not possible to hold them with the right hand, the other endoscopes should be held by the dominant hand while the other hand is manoeuvring the insertion portion. Interestingly, despite the present recommendation to change the endoscope design, the endoscope itself was not considered a risk factor for having injuries in the reviewed studies. The reason may be that the users adapt their position, movement, and endoscopic technique to the endoscope depending on their anthropometrics, and, therefore, they cannot point to one specific endoscopic feature as a risk factor

In the reviewed articles, the main changes were proposed for the GI endoscopes compared to for the others. The suggested endoscope design changes for GI endoscopy are in regard to handle size, weight, shape, and manipulators’ (lever and knobs) force/torque reduction [5,7,8,14,16,20,23,28,29]. Female gastroenterologists expressed the wish of increasing the availability of dial/knob extenders and an optimisation of the endoscope size to better match their smaller hand sizes [28]. Additionally, paediatric GI endoscopes [22], especially colonoscopes [28], being generally thinner, lighter, and more flexible, seemed to be preferred in procedures in adults by professionals with smaller hand sizes. It is positive that 54% of GI endoscopists are willing to train on newly designed endoscopes [29]. In endourology, a reduction in the size of the endoscope and the construction of intuitive tip movement mechanics are proposed [45,46]. Unspecified changes to endoscope design are recommended in nasal endoscopy and bronchoscopy (and GI endoscopy) [22,25,27,33,43].

In general, a reduction in the size and adaptability of the endoscope would favour users, especially females and endoscopists with smaller hand sizes [7,8,16,20,23,28,29,46,63,64]. Human-factors-designed endoscopes would not only favour the ergonomics of the users and their ability to learn endoscopy [63], but would also reduce the duration of the procedures, reported to be longer in bronchoscopy when the professionals have smaller hands (glove sizes smaller than 7.5) [64]. Dial extenders [5,28] or newly designed knobs [65] could improve the reachability in GI endoscopy.

The impact of endoscope weight on the professionals could be already targeted by using an endoscope holder [35], or single-use endoscopes, being significantly lighter than reusable endoscopes [66,67,68]. Using single-use endoscopes has been shown to decrease the forearm muscle activation of the users [69], reducing fatigue, and resulting in a shorter procedure time [66].

To reduce the amount of torque and force necessary to manoeuvre the endoscope, electrically powered wheels could be implemented in new designs of endoscopes [29]. However, adequate maintenance of the equipment can already reduce the force needed to deflect the tip, which increases with use and time [39]. As a result, the force needed to deflect the tip increases, making an already not ergonomically designed device even less ergonomic. In this regard, the use of single-use endoscopes could be considered a solution since the mechanical performance of the device is always that of a new endoscope [66,70].

Finally, additional endoscopic features should ease access to anatomical area and favour the freedom of movement of the users. The use of bronchoscopes with a rotational head simplifies access to the left lobe, by improving the user’s biomechanics [71]. While designing new endoscopes (and medical devices, in general [18]), favouring wireless connection could facilitate the users, independent of the specialties. In addition, implementing the rotatable connection of the umbilical cord to the endoscope processor could improve the manoeuvring of the endoscope [29].

In the future, the further development of robotic-assisted endoscopy could optimise the flow of the procedure and reduce the participation of the endoscopists and, consequently, their physical involvement in the procedure [46]. However, despite the high potential of robotic endoscopy, most robotic platforms are still in development [72,73,74].

#### 4.3.2. Equipment and Operating Room Design

The position of the equipment in the room highly depends on the position of the patient and the one maintained by the endoscopists during the procedure and differs among specialties. In GI endoscopy, professionals rarely perform sitting [7,15], and if they do so, it is during colonoscopy [6]. In nasal endoscopy, the reported position varies among the studies, with prevalence of an even distribution between sitting and standing [32,37,41], while in endourology the professionals sit in most of the cases (76%) [48]. To prevent fatigue of the neck, back, and feet, performing the procedure while sitting is recommend in GI and nasal endoscopy [7,21,30,40].

The adaptability of the equipment allows for the creation of an ergonomic space [4,7,16,25,31,32,35,39,40,43] and, consequently, accommodates all user body structures and different procedure postures. Adjustable monitors and beds are recommended in endourology, as well as GI and nasal endoscopy, to reduce strain on the neck, back, shoulder, and elbow [4,5,8,13,16,18,20,21,27,28,30,32,33,35,39,46,48]. In GI endoscopy [2], the recommendation is to have the table’s height in a neutral position and adjusted between 85 and 120 cm, depending on user height. The monitor should be placed directly in front of the endoscopists, at 52–182 cm to avoid neck rotation and 15–25° off from the horizontal line of the eye’s gaze [75]. These recommendations are based on laparoscopic studies but can easily be transferred to other specialties, as demonstrated by endourology [46]. In addition, in nasal endoscopy, the use of an adjustable chair is recommended [30,32,34,35,39], a good practice that can be transferred to other specialties when involving a sitting position. In general, when sitting is not possible, the use of a cushioned anti-fatigue matt is recommendable [2,7,8,20,27,28]. The foot pedals should be positioned to be within easy reach and fixed so that they are not moved during activation [39].

To allow for the adaptability of the equipment, the room should be large enough to place all the equipment, such as the C-arm fluoroscope, laser lithotripsy machine, towers, ultrasound equipment, monitors, beds, and chairs. Additionally, all the necessary equipment and tools should be placed to be within easy reach [35].

Especially for longer procedures that require the use of fluoroscopy, such as in ERCP, GI stenting, and endourology, the use of a well-fitting [28], two-piece [4,27], and light [44] lead apron has been recommended to reduce back pain.

#### 4.3.3. Ergonomic Best Practice, Workflow, and Institutional Policy Changes

Ensuring an ergonomic posture, i.e., maintaining a neutral neck and back position, with body weight distributed evenly on two feet positioned apart, as well as avoiding hyperextension of the shoulder, back, and knee, is the first ergonomic best-practice recommendation that can help decrease strain in the joints and that is suggested in different endoscopic specialties [18,27,29,34,35,39,41,46]. In GI endoscopy and endourology, the supervision of an ergonomic-trained nurse could be beneficial by verbally advising the endoscopist when the position is not ergonomic [12,13,48]. Additionally, the support of a nurse or fellow is indicated as a measure of ERI prevention by assisting the procedure, helping to move the patient, and applying torque on the insertion portion [8,16,29,35,39]. New technologies, such as wearable sensors, could additionally provide live feedback about the endoscopist’s posture, and a warning when a non-ergonomic position is maintained [76].

A reduction in the procedure volume has been recommended in GI [13,16,18,19,20,25,27] and nasal endoscopy [31,39]. An administrative control of the endoscopy schedule should be implemented, to guarantee a limit of the weekly hours when the endoscopists actively use the endoscopes, planned breaks, and a period of physical rest between procedure days [4,12,39,41]. Microbreaks (also called intraoperative breaks) should be implemented in all endoscopic specialties, despite being recommended only in GI and nasal endoscopy [20,27,34,35]. These are breaks of a few seconds, during which endoscopists can rest the endoscope, shake their hands, and perform some stretching, which are helpful for increasing mental focus, enhancing performance, and reducing the endoscopist’s pain.

Planning an ergonomic time-out between procedures is a suggested preventive measure that can be implemented in all specialties [13,18,19,20,21,24,27,28,35]. It consists of a time before a procedure during which the endoscopist and the staff ensure the adaptation of the height and position of the equipment, the neutralisation of the body posture, and a muscular warm up (stretching and mobility exercises) while indirectly ensuring a physical (and mental) break between procedures. This practice can be easily implemented in all endoscopic specialties during the recommended scheduled break between procedures, guaranteeing an ergonomic environment for the operating staff [4,12,39,41].

Regular physical activity is recommended for all specialties, for both prevention and intervention, to strengthen postural muscles, as well as to increase the endurance that is necessary for long procedures [1,21,47]. Additionally, exercise can help to maintain the bodyweight under control with a consequent reduction in the load on the back and joint [47].

The inconsistency in having received ergonomic training can be related to the lack of ergonomic education of the trainers, and, consequently, 89% of fellows think that teachers should also receive formal training [28]. Receiving regular ergonomic assessments and training is particularly important at the beginning of an endoscopist’s career, during which technical and postural skills are acquired and muscle-learning patterns are trained [51]. Thanks to the development of a more realistic artificial model and to the introduction of virtual reality training as supplements for patient-based endoscopy training, endoscopists could train their posture in a valid and repeatable set-up without risk for patients [77]. It is reassuring that between 45% [21] and 94% [39] of endoscopists are willing to participate in ergonomic training, and up to 88% are motivated to change their practice to prevent ERIs [27].

### 4.4. Limitations

The main limitations of the current review are as follows: Firstly, the reported studies do not always state whether the present injuries are endoscopy-related or not, also because some professionals not only perform endoscopies but also open, laparoscopic, and robotic-assisted surgeries, as in the case of endourologists or otolaryngologists, as well as GI surgeons who are also endoscopists. Therefore, it was not always possible to state whether the ERIs were exclusively caused by performing endoscopies alone. Secondly, in some cases, injuries and pain were considered separately, whereas in other cases there was no distinction between the two. Finally, some publications did not differentiate between flexible and rigid endoscopy. Though rare, rigid (or direct) endoscopies are still performed and, differently from flexible endoscopy, the professionals must look directly through the rigid endoscope, with high strain on the neck and upper back.

The authors of the studies analysed reported various limitations, particularly in relation to sample size [6,9,10,13,14,15,17,18,19,20,23,26,28,32,34,39,40,42,45,48] and methodology, as well as, above all, selection bias [3,5,10,12,13,14,15,16,25,26,27,31,34,36,37,38,39,41,43,44,45,47] and self-reported surveys [10,12,14,16,20,23,26,27,28,41,42,43,46]. Additional methodological limitations highlighted by the authors were, for instance, the provenience of the data only from one site/institute [6,7,22] or the same country [14,16,34], or the lack of inclusion of other specialists [7,15,16,25,26]. For fields such as bronchoscopy, endourology, and nasal endoscopy, the distribution of the use of rigid and flexible endoscopes and of surgery or laparoscopic intervention could not be separated. These procedures require a different set of muscle memory and skills and, therefore, could have affected the ERIs [43].

## 5. Conclusions

The high endoscopy-related musculoskeletal injury incidence reported for endoscopies highlights the need of preventive measures in all specialties. With the increased demand of endoscopies, guaranteeing a safe and ergonomic environment for professionals is mandatory for the healthcare system to increase productivity and decrease career shortening due to work-related injuries, independent of the endoscopic specialty. Additionally, a more ergonomic operating set-up would also translate into a shorter procedure time, and, therefore, being a favourable experience also for the patient.

First, ergonomic training should be promoted during the training of the endoscopy fellows as well as during their careers. The introduction of educational intervention in regard to ergonomics has been demonstrated to have a direct effect on professionals, with a decrease in pain, increased ergonomics, and better comfort [78].

New endoscopes should be developed based on human factors engineering. The ergonomic assessment of endoscopists needs to be considered to ensure a personalised working station with adjustable equipment. Additionally, administrative policies should guarantee that the procedure schedule and volume are planned to include mandatory breaks and rest slots to ensure physical and mental recovery. Microbreaks during a procedure and the assistance of fellows and nurses can benefit an endoscopist’s physical demand.

Future research should concentrate on intervention studies to evaluate in which measure the different ergonomic prevention recommendations are decreasing the ERI incidence. Comparative studies of newly designed equipment should be initiated to assess the potential benefit on the ergonomics of the users compared to existing products. Additionally, biomechanical tests should be favoured to quantify the kinematics, kinetics, and muscle physiology of endoscopists in the operating room.

## Figures and Tables

**Table 1:** PECO framework (ERIs: endoscopic-related injuries).

Acronym	Definition	Motivation	Research Question
P	Population	Gain insights about demographic, work, and ERI characteristics of the endoscopists	Which are the demographic and work characteristics and the ERI rate of the professionals of different endoscopic specialties?
E	Exposure	Analyse the risk factors that are leading to ERIs in the different endoscopic specialties	Which risk factors are leading to ERIs in the different endoscopic specialties?
C	Comparison	Individuate analogies and differences of the ERI characteristics of the different endoscopic specialties	Which differences and analogies can be made regarding ERI impact, ergonomics, and risk factors in the different endoscopic specialties?
O	Outcome	Provide recommendations to reduce ERIs among endoscopists	Which preventive measures could reduce the ERI rate among endoscopists?

## Data Availability

The data presented in this study are available on request to the corresponding author.

## Appendix A

**Table A1:** Study (first author, year of publication, and reference), aim, type of survey (printed/online), and number (n) of questions and of recipients of the reviewed articles (ERIs: endoscopy-related injuries; GI: gastrointestinal; ENT: ear–nose–throat endoscopy).

Study	Aim	Type: Questions (n)	Recipients (n)
**Gastrointestinal endoscopy**			
Buschbacher, 1994 [3]	To determine what kinds of overuse syndromes professionals suffer from because of carrying out endoscopic procedures in the United States of America	Printed: -	367
O’Sullivan, 2002 [4]	To examine the current practices of duodenoscopists and the prevalence of ERIs in Canada	Printed: -	1662
Liberman, 2005 [5]	To identify ERIs specific to physicians routinely performing colonoscopies, and to identify prevention strategies	Online: -	2173
Byun, 2008 [6]	To assess the prevalence, severity, risk factors, and clinical impact of ERIs among GI endoscopists in Korea	-: -	55
Hansel, 2009 [7]	To identify the frequency and significance of ERIs compared with a similar group of non-procedure-oriented internal medicine physicians in the United States of America	Online: -	345
Kuwabara, 2011 [8]	To investigate the frequency and prevention of ERIs in Japanese GI endoscopists and non-endoscopist physicians	-: -	448
Drysdale, 2013 [9]	To discover the prevalence of upper-extremity ERIs in endoscopy nurses in Canada and factors in the workplace that may be associated with increased risk, and to determine whether certain subgroups may be at increased risk	-: -	220
Ridtitid, 2015 [10]	To define the prevalence and types of ERIs in the current era of high-volume endoscopies with advanced therapeutics and to evaluate endoscopist and practice characteristics associated with these injuries in the United States of America	Online: 25	5239
Ahmed, 2016 [11]	To ascertain the value of video training on occupational injury and the best practices to minimise injury from performing endoscopies in the United States of America	Online: 29	-
Austin, 2019 [12]	To determine the rate and types of ERIs, to evaluate risk factors, and to evaluate program directors’ knowledge of the prevalence of ERIs in the United States of America	Online: 29	216
Villa, 2019 [13]	To internationally investigate the prevalence of ERIs among GI fellows	Online/printed: 22	217
Han, 2020 [14]	To internationally assess the prevalence and types of ERIs in third-space endoscopists and its impact on clinical practice	Online: 22	110
Morais, 2020 [15]	To evaluate the prevalence, type, and impact of ERIs in regular and labour activity, and risk factors among Portuguese endoscopists	Online: 39	705
Al-Rifaie, 2021 [16]	To internationally explore the prevalence and range of colonoscopy-related ERIs	Online: 20	1825
Campbell, 2021 [17]	To assess the prevalence of ERIs associated with endoscopic retrograde cholangiopancreatography in the United States of America and to identify risk factors that may help direct future device and protective equipment development	Online: 23	3276
Kamani, 2021 [18]	To assess the risk factors of ergonomic injuries among endoscopists and non-endoscopists in Pakistan	-	-
Matsuzaki, 2021 [19]	To evaluate the prevalence of ERIs and identify the risk factors for developing ERIs, focusing on procedure time among endoscopists in Japan	Online: 50	213
Pawa, 2021 [20]	To assess the prevalence of self-reported ERIs, patterns of injury, and endoscopist knowledge of preventative strategies in the United States of America	Online: 38	15,868
Bessone, 2022 [21]	To investigate the correlation between the anthropometrical characteristics and the occurrence and location in GI endoscopies internationally	Online: 32	-
Miller, 2022 [22]	To use structured objective procedural data to analyse ERI prevalence, type, and distribution, and to evaluate whether endoscopic volume and efficiency impact ERIs in the United States of America	Online: 11	179
Pawa, 2022 [23]	To assess the rate of self-reported ERI occurrence, patterns of injury, and knowledge of preventive strategies in the United States of America	Online: 38	15,868
Shah, 2022 [24]	To document the prevalence of ERIs, and awareness and practices of ergonomics by endoscopists and ancillary staff in Pakistan	Printed: 11	-
Sturm, 2022 [25]	To explore the risk factors, impact, and prevalence of musculoskeletal disorders in practicing German endoscopists	Online: 24	-
Costello, 2023 [26]	To internationally measure the perceived incidence of staff ERIs and patient complications among endoscopy nurses and their associations with institutional ergonomic policies	Online: 7	278
Ruan, 2023 [27]	To examine the prevalence, nature, and impact of ERIs in paediatric endoscopic practice and assess attitudes towards ergonomic training needs internationally	Printed: 27	233
Suhail, 2023 [28]	To examine sex differences in the prevalence of ERIs and ergonomic training during GI fellowship in the United States of America	Online: 56	704
Bhatt, 2024 [29]	To analyse differences in endoscopy styles, technique preferences, and ERIs between female and male gastroenterologists in the United States of America	Online: 34	814
**Nasal endoscopy**			
Babar-Craig, 2003 [30]	To determine the point prevalence of neck and back pain in ENT consultants in the United Kingdom and which sub-specialities were most at risk	Printed: -	558
Cavanagh, 2012 [31]	To investigate the prevalence of workplace-related discomfort and injury and to assess professionals’ knowledge of workplace ergonomic principles in the United States of America	Online: 20	229
Little, 2012 [32]	To define the prevalence, quality, and severity of physical symptoms that otolaryngologists experience in the United States of America	-: 25	-
Rimmer, 2016 [33]	To determine the prevalence of musculoskeletal symptoms among ENT surgeons in Europe	Online: 20	2235
Vjendren, 2016 [34]	To investigate the prevalence of various ERIs among ENT doctors in the United Kingdom	Online: -	1344
Dabholkar, 2017 [35]	To investigate the ERI prevalence in India and to analyse the risk factors encountered in the operation and outpatient rooms	Printed: -	150
Wong, 2017 [36]	To identify and evaluate the characteristics of musculoskeletal symptoms in otolaryngology residents in the United States of America	Online: -	-
Bolduc-Bégun, 2018 [37]	To identify ERI symptoms and determine their prevalence in Canada and to establish potential associations between ERIs and demographic variables	Online: -	595
Ho, 2018 [38]	To assess ERI symptoms among otolaryngologists across subspecialties and to quantify the understanding and application of ergonomic principles in the operating room in the United States of America	Online: -	3006
Lobo, 2019 [39]	To determine the prevalence of ERI symptoms in otolaryngology and head and neck surgery specialists and trainees in Spain and to identify risk factors. To identify and measure the effect that physical exercise and the level of physical activity could have on the appearance of ERIs. To assess the individual perception of the magnitude of this problem and the level of knowledge of the principles and recommendations in studies and research in the field of surgical ergonomics	Online: -	2450
Vaisbuch, 2019 [40]	To evaluate the severity of ERIs in ENT endoscopy	Online: 14	70
Dahmash, 2020 [41]	To identify the prevalence of ERIs and risk factors among otorhinolaryngologists resident in Saudi Arabia	Printed: -	66
Aaronson, 2023 [42]	To identify surgeon preferences, when practices become norms, and whether procedure positions are associated with surgeon discomfort or ERIs in seven different operating procedures in paediatric otolaryngology in the United States of America	Online: 23	178
**Bronchoscopy**			
Gilbert, 2013 [43]	To define the impact that ERIs are currently having on practising pulmonologists in the United States of America and identify modifiable factors that can be evaluated in future studies	Online: 25	199
**Endourology**			
Elkoushy, 2011 [44]	To assess the compliance of endourologists with radiation safety measures and to determine the prevalence of orthopaedic complaints	Online: 7	-
Healy, 2011 [45]	To determine the prevalence and causes and risk factors of hand problems among endourologists who routinely perform flexible ureteroscopy compared with controls internationally	Online: -	1178
Tjiam, 2014 [46]	To provide an international overview of the type and frequency of ERIs among urologists and their knowledge about ergonomics	Online/printed: 19	-
Lloyd, 2019 [47]	To factor out the contribution of different surgical types, age, volume, and locality, and to identify other predictive factors in the nonwork life of these practitioners, in a global setup	Online: 14	-
Omar, 2020 [48]	To survey the prevalence and possible causes of musculoskeletal disorders among endourologists performing transurethral resections of the prostate or laser prostatectomy globally	Online: -	3000

**Table A2:** Demographic, anthropometric, and work data in the different fields of endoscopy (BMI: body mass index; ♂: male; ♀: female; size: XS: extra-small; S: small; M: medium; L: large; XL: extra-large; EGD: esophagogastroduodenoscopy; ERCP: endoscopic retrograde cholangiopancreatography; EUS: endoscopic ultrasound; h: hours).

Study	Responses (Rate)	Male	Age (Years)	Anthropometric Data	Glove Size	Right-Hand	Experience (Years)	Active	Endoscopic Volume	Others
**Gastrointestinal endoscopy**										
[3]	265 (72%)	95% ^1^	48 ± 9 ^1^	-	-	83%	>6 months: 100%	-	h/week: 0–10: 44% ^2^ 11–20: 48% ^2^ 21–30: 7% ^2^ 31–40: 1% ^2^	-
[4]	114 (70% ^2^)	-	-	-	-	-	15 ± 7 ^6^	100%	ERCP/year: 193 ± 127 ^1,6^	Physically active: 84%
[5]	608 (28% ^1^)	89% ^1^	48 ± 10 ^1^	-		74% ^1^	15 ± 9 ^1^	96%	Colonoscopies/day: 7 ± 4 Colonoscopy days/week: 2 ± 2 ^1^	-
[6]	55 (100%)	67% ^2^	39 (28–47)	-	-	-	3 ^2,4^	100%	h/week: 20 ± 8 ^1^ Endoscopies/month: 270 ± 153 ^1^	-
[7]	72 (51% ^2^)	83% ^1,3^	25–35: 6% ^2,3^ 36–45: 48% ^2,3^ 46–55: 37% ^2,3^ 56–65: 10% ^2,3^ >66: 0% ^2,3^	Height ^4^ (cm): 1.77 ± 0.09 ^1^ Weight (kg): 77 ± 13 ^1^ BMI (kg/m^2^): 24 ± 3 ^1^	M: 32% ^1,4^ L: 38% ^1,4^	87% ^1^	≤15: 58% ^2,4^ 16–20: 20% >21: 23% ^1,4^	97%^1^	≤10 half-days/month: 51% ^1^ ≥21 half-days/month: 14% ^1^ EGD/week: 13 ± 10 ^1^ Colonoscopies/week: 16 ± 11 ^1^ ERCP/week: 10 ± 8 ^1^	Experience in ERCP/EUS: 7% ^1^ Physical activity ^3^: Sedentary: 0% ^1^ Mild: 16% ^1^ Moderately: 70% ^1^ Heavy: 14% ^1^
[8]	310 (69% ^1^)	86% ^1,3^	41 ± 7 ^1,3^	Height (m): 1.68 ± 0.7 ^1,3,4^ Weight (kg): 66 ± 11 ^1,3^ BMI (kg/m^2^): 23 ± 2 ^1,3^	-	94% ^1^	16 ± 8 ^1,3^	-	EGD/week: 15 ± 9 ^1^ Colonoscopies/week: 7 ± 4 ERCP/week: 2 ± 2 ^1^ EUS/week: 2 ± 1 ^1^	Physical activity ^3^: Mild: 69% Moderate: 26% Heavy: 3%
[9]	147 (67% ^2^)	-	50 ± 8 ^1^	Height ^4^ (m): 1.64 ± 0.66 ^1,3^ Weight ^4^ (kg): 69 ± 14 ^1,3^ BMI (kg/m^2^): 26 ± 4 ^1^	-	-	11 ± 8 ^1^	-	-	Nurses (100%)
[10]	684 (13%)	88% ^1^	51 ± 11 ^1^	Height (m): 1.76 ± 0.10 Weight (kg): 83 ± 35 ^1^	-	90% ^1^	≤15: 43% ^1^ 16–30: 37% ^1^ >30%: 21% ^1^	97% ^1^	0–20 procedures/week: 27% ^1^ 21–40 procedures/week: 52% ^1^ 41–60 procedures/week: 18% ^1^ >60 procedures/week: 3% ^1^ 0–15 h/week: 36% ^1^ 16–30 h/week: 55% ^1^ >30 h/week: 10% ^1^	Physical activity: Mild: 15% ^1^ Moderate: 44% ^1^ Heavy: 41% ^1^
[11]	58 (-)	57% ^1^	-	--	-	-	Fellowship: 1st year: 40% ^1^ 2nd year: 22% ^1^ 3rd year: 36% ^1^	-	>150 EGDs: 60% ^1^ >150 colonoscopies: 57% ^1^	-
[12]	165 (76% ^2^)	65% ^2^	28–30: 23% 31–35: 67% 36–40: 7% 41–44: 2%	-	-	90%	Year of training: 1st: 33% 2nd: 30% 3rd: 33% 4th: 4%	-	No procedure/half-day: 23% 3–4 procedure/half-day: 16% 5–6 procedure/half-day: 41% 7–8 procedure/half-day: 13%	Recreational activity: Light: 39% ^1^ Moderate: 20% ^1^ Heavy: 36% ^1^
[13]	156 (72% ^2^)	65%	25–30: 15% ^1^ 31–35: 64% ^1^ 36–40: 20% ^1^ >40: 1% ^1^	-	-	-	Fellowship 1st year: 33% ^1^ 2nd year: 32% ^1^ 3rd year: 34% ^1^ Other: 1% ^1^	-	-	Experience in ERCP: 28% ^1^
[14]	319 (18% ^2^)	98% ^1^	46 ± 7 ^2^	-	7.6 ± 0.5	82% ^1^	12 ± 9 ^1^	100%	-	Experience in ERCP: 89% ^1^ Regular physical activity: 91% ^1^ Hours spent exercising/week: 3 ± 2 ^1^
[15]	171 (24% ^1^)	45% ^1^	36 (26–78)	Height (cm): 170 (150–190) Weight (kg): 65 (47–92) BMI (kg/m^2^): 23 ^1^ (17.6–31.6)	S: 32% ^1^ M: 46% ^1^ L: 20% ^1^ XL: 2% ^1^	92% ^1^	9	98% ^1^	Procedure h/week: 25 (3–52)	Physical activity: Sedentary: 28% ^1^ Light: 47% ^1^ Moderate: 22% ^1^ Vigorous: 3% ^1^
[16]	319 (18% ^1^)	68% ^1^	20–30: 1% ^1^ 31–40: 20% ^1^ 41–50: 42% ^1^ 51–60: 28% ^1^ >60: 9% ^1^	-	-	-	0–10: 38% ^2^ >10: 62% ^1^	-	<150 colonoscopies/year: 17% ^1^ >150 colonoscopies/year: 83% ^1^ <6 h/week: 14% ^1^ >6 h/week: 86% ^1^	Gastroenterologists (68% ^1^), nurses (25% ^1^) and surgeons (7% ^1^)
[17]	203 (6% ^1^)	95% ^1^	-	-	S: 4% ^2^ M: 35% ^2^ L: 48% ^2^ XL: 12% ^2^	-	≤15: 43% ^2^ 16–20: 10% ^2^ >20: 46% ^2^	-	0–100 ERCP/year: 48% ^2^ 101–200 ERCP/year: 26% ^2^ 201–500 ERCP/year: 21% ^2^ >500 ERCP/year: 4% ^2^ 0–100 not ERCP/year: 2% ^2^ 101–200 not ERCP/year: 3% ^2^ 201–500 not ERCP/year: 12% ^2^ >500 not ERCP/year: 83% ^2^	-
[18]	92 (-)	95% ^1,3^	44 ± 8 ^1,3^	Weight (kg): 80 ± 13 ^1^ Height (m): 1.76 ± 0.12 BMI (kg/m^2^) ^1,3^: 26 ± 4	6.5: 2% ^1,6^ 7.0: 24% ^1,6^ 7.5: 52% ^1,6^ 8.0: 21% ^1,6^ 8.5: 2% ^1,6^	92% ^1^	≤20 ^6^: 95% ^1^ >20 ^6^: 5% ^1^	-	<20 procedures/week ^6^: 55% ^1^ ≥20 procedures/week ^6^: 45% ^1^ ≤5 h/week ^6^: 86% ^1^ >5 h/week ^6^: 14% ^1^	Experience in ERCP/EUS ^6,7^: 53% ^1^
[19]	110 (52% ^2^)	89% ^1^	<29: 1% ^1^ 30–39: 56% ^1^ 40–49: 19% ^1^ 50–59: 21% ^1^ >60: 4% ^1^	BMI (kg/m^2^): 23 ± 3 ^1^	-	96% ^1^	≤15: 65% ^2^ ≥16: 35% ^1^ No data: 1% ^1^	100%	h/week: 55 ± 11 ^1^ Procedure ^7^ (min/month): Upper GI endoscopy: 380 ± 229 ^1^ Lower GI endoscopy: 565 ± 477 ^1^ ERCP: 207 ± 305 ^1^	Sedentary time (h/day): 4 ± 3 ^1^
[20]	1698 (11% ^2^)	66% ^1^	52 ± 12 ^1^	Weight ^4^ (kg): 77 ± 15 ^1^ Height ^4^ (m): 1.73 ± 0.10 ^1^ BMI (kg/m^2^): 26 ± 4 ^1^	♀: XS-M: 97% ^1^ ♂: L to XL: 73% ^1^	-	21 ± 12 ^1^	92% ^1^	-	-
[21]	204 (-)	78% ^1^	<34: 9% ^1^ 35–44: 34% ^1^ 45–54: 37% ^1^ 55–64: 18% ^1^ >65: 3% ^1^	Height (m): 1.50–1.59: 8% ^1^ 1.60–1.69: 21% ^1^ 1.70–1.79: 44% ^1^ 1.80–1.89: 21% ^1^ 1.90–1.99: 6% ^1^ Weight (kg): <50: 3% ^1^ 50–59: 9% ^1^ 60–69: 24% ^1^ 70–79: 30% ^1^ 80–89: 22% ^1^ 90–99: 8% ^1^ >100: 8% ^1^	5.5: 1% ^1^ 6.0: 4% ^1^ 6.5: 21% ^1^ 7.0: 18% ^1^ 7.5: 31% ^1^ 8.0: 18% ^1^ 8.5: 4% ^1^ 9.0: 3% ^1^	92% ^1^	≤15: 49% ^2^ 16–25: 33% ^1^ 26–35: 13% ^1^ >35: 5% ^1^	-	≤10 procedures/week: 18% ^2^ 11–20 procedures/week: 25% ^2^ >20 procedures/week: 74% ^2^ <10 h/week: 25% ^2^ 11–20 h/week: 40% ^2^ >20 h/week: 35% ^2^	Use of corrective lenses: 40% Experience in ERCP: 73% ^1^ Physical activity (h/week): 0: 18% ^1^ 1–2: 27% ^1^ 3–5: 32% ^1^ 6–9: 16% ^1^ ≥10: 7% ^1^ Leisure activity involving the use of fingers: 33% ^1^
[22]	64 (36% ^2^)	72% ^1^	44 ± 11 ^1^	Height ^4^ (m): 1.76 ± 0.11	M-L	80% ^1^	19 ± 11 ^1^	100%	Procedures/week: 15 ± 8 ^2^ EGD/week: 5 ± 3 ^2^ Colonoscopies/week: 7 ± 5 ^2^	Experience in ERCP: 8% Experience in EUS: 9%
[23]	168 (1% ^1^)	51% ^1^	32 ± 3 ^1^	Height^4^ (m): 1.71 ± 0.10 BMI (kg/m^2^): 24.5 ± 3.5 ^1^	♀: 6.0 ♂: 7.5	-	2 ± 1 ^1^	-	♀/♂: >10 EGD/week: 67% ^1^/45% ^1^ >10 colonoscopies/week: 66% ^1^/54% ^1^ >10 ERCP/week: 2% ^1^/4% ^1^	-
[24]	56 (-)	70% ^1^	35 ^1^	-	-	88% ^1^	<5: 49% ^1^ >5: 51% ^1^	-	Procedures/week: 64 ^1^	Endoscopists (23% ^1^), nurses (27% ^1^), and endoscopy technicians (34% ^1^) Physical activity (h/week): 0: 41% ^1^ <2.5: 23% ^1^ 2.5: 9% ^1^ >2.5: 27% ^1^
[25]	151 (-)	73% ^1^	50 ± 10 ^1^	Height ^4^ (m): 1.78 ± 0.09 ^1^ Weight (kg): 79 ± 13 ^1^ BMI (kg/m^2^): 25 ^1^	S: 15% ^1^ M: 24% ^1^ L: 44% ^1^ XL: 14% No data: 4% ^1^	84% ^1^	21 ± 10 ^1^	100%	Procedures/week: 86 ± 38 ^1^	Physical activity: None: 19% ^1^ 1–2 times/week: 52% ^1^ 3–4 times/week: 20% ^1^ Daily: 5% ^1^ No data: 4% ^1^
[26]	185 (67% ^1^)	-	-	-	-	-	-	-	-	Nurse (75% ^1^)
[27]	146 (63% ^1^)	45% ^1^	-	BMI (kg/m^2^): <18.5: 3% ^1^ 18.5–24.9: 57% ^1^ 25–29.9: 28% ^1^ >30: 12% ^1^		93% ^1^	Fellow: 34% ^1^ 1–15: 61% ^2^ 16–20: 6% ^1^ >20: 13% ^1^	100%	<10 h/week: 85% ^2^ 10–20 h/week: 13% ^2^ >20 h/week: 2% ^2^ >10 EGD/week: 6% ^1^ >10 colonoscopies/week: 0% ^1^ >10 advanced endoscopy/week: 1% ^1^ <10 procedures/week: 83% ^2^ >10 procedures/week: 17% ^2^	Physical activity: Light: 17% ^1^ Moderate: 42% ^1^ Heavy: 41% ^1^
[28]	236 (34% ^1^)	52%	<34: 86% ^1,7^ 35–50: 14% ^1^ >40: 0% ^1^	Height ^4,5^ (m) ♀/♂: 1.52–1.60: 38%/- 1.63–1.68: 46%/- 1.70–1.75: 25%/41% 1.78–1.83: -/44% 1.85–1.93: -/24%	♀/♂ ^5^: 6.0: 29%/- 6.5: 55%/- 7.0: 15%/36% 7.5: -/60% 8.0: -/16%	-	Fellowship: 1st year: 34% ^1^ 2nd year: 33% ^1^ 3rd year: 28% ^1^ Advanced: 6% ^1^	-	<10 h/week: 24% ^1^ 10–20 h/week: 47% ^1^ 21–30 h/week: 21% ^1^ 31–40 h/week: 6% ^1^ >40 h/week: 3% ^1^	-
[29]	107 (13% ^2^)	62% ^1^	<40: 42% ^1^ 40–60: 42% ^1^ >60: 16% ^1^	Height ^4^ (m): <1.57: 8% ^1^ 1.57–1.73: 40% ^1^ >1.73: 51% ^1^	XS: 1% ^1^ S: 20% ^1^ M: 42% ^1^ L: 28% ^1^ XL: 9% ^1^	-	≤10: 59% ^2^ >10: 41% ^1^	100%	<20 procedures/week: 36% ^1^ 20–40 procedures/week: 46% ^1^ >40 procedures/week: 19% ^1^	Use of corrective lenses: 9% ^1^
**Nasal endoscopy**										
[30]	325 (58%)	-	47	-	-	-	12	-	-	-
[31]	100 (44% ^1^)	85%	53 ± 8 ^1^	Height (m): 1.76 ± 0.11 ^2^	-	-	21 ± 9 ^1^	100%	-	-
[32]	62 (-)	77%	36 (28–64)	Height ^4^ (m): 1.78	-	88%	≤15: 77% ^2^ >15: 23%	-	-	-
[33]	250 (11% ^1^)	79% ^1^	30–40: 20% 40–50: 35% 50–60: 32% >60: 13%	-	-	-	1–10: 40% ^2^ 10–20: 34% 20–30: 26%	100%	<50 procedures/year: 32% ^1^ 50–100 procedures/year: 45% ^1^ >100 procedures/year: 23% ^1^	-
[34]	323 (24% ^2^)	-	-	-	-	-	19 ^1^ ≤10: 18% ^2^ 11–20: 33% ^2^ 21–30: 22% ^1^ >30: 17% ^1^ N/A: 10% ^1^	-	-	-
[35]	73 (49% ^2^)	63% ^1^	37 ± 11 ^1^	Height ^4,6^ (m): 1.66 ± 0.08 ^1^ Weight ^4,6^ (kg): 72 ± 15 ^1^	7 ^6^	-	11 ± 9 ^1^	100%	Surgeries/week: 5 ± 2 ^1^ Surgery h/week: 13 ± 12 ^1^	Physically active ^6^: 51% ^1^
[36]	141 (35% ^1^)	55%	30 ^1^	Height (m): 1.74 Weight (kg): 70 ^1^ BMI (kg/m^2^): 23 ^1^	-	96%	PGY-1: 15% ^1^ PGY-2: 18% ^1^ PGY-3: 18% ^1^ PGY-4: 25% ^1^ PGY-5: 18% ^1^ PGY > 5: 4% ^1^	-	-	-
[37]	137 (23%)	79%	46	Height (m): 1.75 Weight (kg): 77 BMI (kg/m^2^): 25 ^1^	-	90%	≤15: 58% ^2^ 15–25: 21% >25: 21%	-	Procedure h/week: 0–4: 8% 4–8: 32% 8–12: 36% >12: 24% Procedures/week: <5: 25% 5–8: 45% >8: 29%	Physical activity (h/week): <3: 50% >3: 50%
[38]	377 (13% ^1^)	73% ^1^	42 ± 14 ^1^	Height ^4^ (m): 1.78 ± 0.09 Weight ^4^ (kg): 82 ± 12	-	91% ^1^	Fellow: 46% ^1^ 0–5: 13% ^1^ >5: 42% ^1^	-	-	-
[39]	403 (17% ^1^)	52% ^1^	45 ± 11 ^1^	Weight (kg): 72 ± 11 ^1^ BMI (kg/m^2^): 25 ± 3 ^1^	-	-	17 ± 11 ^1^	-	Procedures/week: 10 ± 5 ^1^ h/week: 29 ± 17 ^1^	Physical active (h/week): 3 ± 3 ^1^
[40]	48 (69% ^1^)	69% ^1^	-	-	-	-	≤10: 48% ^2^ 11–20: 19% ^1^ 21–30: 21% ^1^ >30: 13% ^1^	-	-	-
[41]	45 (68% ^1^)	73% ^1^	25–27: 36% ^1^ 28–30: 64% ^1^	Height ^4^ (m): 1.70 ± 0.08 ^1^ Weight (kg): 74 ± 15 ^1^ BMI (kg/m^2^): 25 ± 4 ^1^	-	89% ^1^	2nd year: 24% ^1^ 3rd year: 24% ^1^ 4th year: 33% ^1^ 5th year: 18% ^1^	100%	Procedures/week: 2 ± 1 ^1^ h/week: 8 ± 4 ^1^	Physical activity (days/week) 0: 40% ^1^ 1: 18% ^1^ 2: 22% ^1^ 3: 20% ^1^
[42]	69 (39% ^2^)	-	-	-	-	-	1–10: 42% ^2^ 11–20: 17% 21–30: 26% >31: 15%	-	<20 procedures/month: 6% 21–40 procedures/month: 15% 41–60 procedures/month: 32% 61–80 procedures/month: 19% >81 procedures/month: 29%	-
**Bronchoscopy**										
[43]	160 (80% ^2^)	86%	45 ± 9	Height ^4^ (m): 1.75 ± 0.10	7.5 ± 0.5	-	11 ± 10	-	-	-
**Endourology**										
[44]	134 (-)	-	<40: 25% ^1^ 40–60: 63% ^1^	-	-	-	<10: 29% ^1^ 10–20: 37% ^1^ >20: 34% ^1^	-	≤1 day/week: 18% ^1^ 2–3 days/week: 66% ^1^ 4–5 days/week: 16% ^1^ Ureteroscopy/year: 60 Percutaneous nephrolithotomy/year: 25 Retrograde urography/year: 40 Shock-wave lithotripsy/year: 27	-
[45]	196 (17% ^2^)	-	-	-	-	87%	13 ^1^	-	Procedures/week: 5 ^1^	-
[46]	285 (-)	93% ^2^	46 ± 8 ^1^	Height ^4^ (m): 1.79 ± 0.08	7.5 ± 0.5	-	13 ± 8 ^1^	-	h/week: 8 ± 7 ^1^	-
[47]	701 (-)	80% ^2^	-	Height (m): 1.75 ± 0.08 Weight (kg): 78 ± 14 ^1^ BMI (kg/m^2^): 25 ± 4 ^1^	-	-	-	-	-	Physical activity (times/week): 0–1: 42% ^1^ 2–3: 34% ^1^ >4: 24% ^1^
[48]	121 (4% ^2^)	98% ^2^	25–34: 5% ^2^ 35–44: 38% ^2^ 45–54: 31% ^2^ 55–64: 17% ^2^ >64: 8% ^2^	BMI (kg/m^2^): 27 ± 5 ^1,6^	-	92% ^1^	≤15: 58% ^2^ >15: 42% ^2^	-	h/week ^6^: 16 ± 10 ^1^	Physical activity ^6^ (h/week): 4 ± 3 ^1^ Wearing eyeglasses or contact lenses: 65% ^1^

^1^ Value rounded for conciseness. ^2^ Calculated by the reviewers based on the published data of the article. ^3^ Value specific for endoscopists. ^4^ Data converted from non-metric, or non-standard system to metric system. ^5^ Data retrieved from graph. ^6^ Among physicians with endoscopy-related injuries. ^7^ Additional data reported in the original article.

**Table A3:** Endoscopy-related injuries (ERIs) in the different fields of endoscopy (♂: male; ♀: female; carpal tunnel syndrome (CTS); De Quervain tenosynovitis (DQT); non-steroid anti-inflammatory drug (NSAID); operating room (OR)). Additional notes are reported in italics.

Study	All/♀/♂ Rate	Location	Type	Leave	Treatment	Practice Modification Due to ERIs	Others/Notes
**Gastrointestinal endoscopy**							
[3]	44% ^1^/-/-	Low back (27%), thumb (19%), shoulder (19%), elbow (15%), hand (14%), neck (13%) *Indicated as pain location*	Hand numbness (12%), CTS (6%) *Pain distributed as reported in “Location”*	-	No treatment (50% ^1^), medication (18% ^1^), physiotherapy (17% ^1^), rest (8% ^1^), splint (5% ^1^) Surgery (3% ^1^)	-	-
[4]	67%/-/-	Back (57%), neck (46%), hand (36%), shoulder (16%), elbow (8%) *Indicated as pain location*	*Pain distributed as reported in “Location”*	-	No treatment (45%), medication (36%), physiotherapy (15%), massage (13%), chiropractic (11%), rest (11%)	18% made a modification (ERCP technique modification)	58% reported two or more ERIs. 74% ERCP-related
[5]	37% ^1^/-/-	Foot (34% ^1^), neck (29% ^1^), right hand (24% ^1^), back (23% ^1^), right finger (20% ^1^), left finger (17% ^1^), left hand (17% ^1^), left thumb (14% ^1^), right wrist (11% ^1^), right elbow (10% ^1^), right thumb (9% ^1^), right shoulder (9% ^1^), left shoulder (6% ^1^), left elbow (4% ^1^), left wrist (3% ^1^), knee (2% ^1^), hip (1%)	CTS (5% ^1^) *Pain distributed as reported in “Location”*	1% ^2^	NSAID (37% ^2^), physiotherapy (9% ^2^), splint (5% ^2^), thermal treatment (4% ^2^), steroid injection (2% ^2^) Surgery: 3% ^2^ 21% ^2^ sought medical care	-	Leave due to ERIs: 2–20 days
[6]	89% ^1^/61% ^2^/41% ^2^	Left finger (16% ^1^), left shoulder (15% ^1^), right wrist (15% ^1^), left wrist (9% ^1^), right shoulder (7% ^1^)	-	-	NSAID (29% ^1^) 14% ^1^ sought medical advice	16% ^1^ modified their practice (procedure reduction, stretching, exercising, rest)	Number of ERI location: 4 ± 3 ^1^
[7]	74% ^1^/-/-	Thumb (19% ^1^), low back (19% ^1^), hand/finger (17% ^1^), neck (10% ^1^)	Pain (31% ^1^), aching (15% ^1^)	13% ^1^	36% ^1^ sought medical care	69% ^1^ made a modification (stretching, procedure reduction, floor mat, and adjustable bed)	-
[8]	43% ^3^/-/-	Back (62% ^2^), neck (22% ^2^), right shoulder (22% ^2^), left thumb (20% ^2^), left shoulder (19% ^2^), left wrist (16% ^2^), right hand/fingers (6% ^2^), left hand/fingers (5% ^2^), right thumb (4% ^2^), right wrist (4% ^2^) *Indicated as pain location*	*Pain distributed as reported in “Location”*	-	26% sought medical care	12% made a modification (stretching, increasing breaks, athletic shoes, procedure reduction)	-
[9]	-/-/-	-	-	-	Analgesics: 42% ^1^ occasional use, 12% ^1^ regular use 60% ^1^ sought medical care	-	-
[10]	53% ^1^/33% ^1^/87% ^1^	Thumb (28% ^1^), neck/upper (29% ^1^), lower back (19% ^1^), elbow (11% ^1^), shoulder (10% ^1^), hand (10% ^1^) *Indicated as pain location*	CTS (6% ^1^), hand numbness (4% ^1^) *Pain distributed as reported in “Location”*	19% ^1^	Medications (57% ^1^), physiotherapy (45% ^1^), rest (34% ^1^), steroid injection (27% ^1^), splint (23% ^1^) Surgery: 13% ^1^	55% ^1^ made a modification (stretching, increasing breaks, orthopaedic shoes, procedure reduction, floor mat, and adjustable table)	-/24% ^1^/88% possibly ERIs
[11]	78% ^1^/-/-	Wrist (82%), thumb (46%), neck (40%)	-	2% ^1^	No treatment (78%)	-	-
[12]	20% ^1^/34% ^1^/12% ^1^	Thumb/hand (64%), neck/upper back (23%), shoulder (18%), low back (18%), elbow (14%) *Indicated as pain location*	Hand numbness/CTS ^5^: 16% *Pain distributed as reported in “Location”*	11%	Rest (39%), medications (36%), no treatment (27%), splint (23%), physiotherapy (18%), steroid injections (5%), massage (2%), icing (2%) Surgery: 2% 20% sought medical care	11% made a modification (stretching, technique modification, brace, increasing breaks, procedure reduction, orthopaedic shoes, hand exercise, larger gloves)	7% ^1^ possible ERIs
[13]	47% ^1^/56% ^1^/43% ^1^	Right wrist (53%), left thumb (48%), back (31%), neck (22%)	-	3%	NSAID (42% ^2^), splint (3% ^2^)	65% ^1^ made a modification	Leave due to ERIs: 0 day: 96% ^2^ 1–3 days: 4% ^2^
[14]	69%/-/-	Shoulder (42% ^1^), back (38% ^1^), neck (33% ^1^), wrist (24% ^1^), hand (18% ^1^), thumb (16% ^1^)	-	2% ^1^	NSAID (49% ^1^), massage (27% ^1^), pain medications (20%), splint/braces (11% ^1^), chiropractic (7% ^1^), injections (2% ^1^), physiotherapy (2% ^1^) Surgery: 2% ^1^	2% ^1^ reduction in clinical schedule	-
[15]	70% ^1^/60% ^1^/40% ^1^	Neck (30% ^1^), thumb (29% ^1^), shoulder (28% ^1^), wrist (27% ^1^), lower back (18% ^1^), upper back (15% ^1^), hand (15% ^1^), elbow (9% ^1^) *Indicated as pain location*	Hand numbness (12% ^1^) *Pain distributed as reported in “Location”*	10% ^1^	NSAID (57% ^1^), physiotherapy (30%), rest (29% ^1^), no treatment (25% ^1^), paracetamol (18% ^1^), corticosteroid injection (13% ^1^), splint (10% ^1^) Surgery: 2% ^1^	61% ^1^ made a modification (procedure reduction, stretching, increasing breaks, use adjustable bed, orthopaedic shoes)	Leave due to ERIs: 30 days
[16]	78% ^2^/84% ^2^/69% ^2^	Lower back (37% ^1^), neck (35% ^1^), left thumb (34% ^1^), right shoulder (27% ^1^), right wrist (25% ^1^), upper back (20% ^1^), right hand (19% ^1^), right thumb (18% ^1^) ^5^	CTS (6% ^1)^ *Pain distributed as reported in “Location”*	10% ^1^	Physiotherapy (43% ^2^), medications (28% ^2^), rest (17% ^2^), splint (12% ^2^), steroids (10% ^2^) Surgery: 4% ^2^	31% ^1^ made a modification (stretching, rest, adjusting ergonomics, regular massages, splints and supporting devices, alternating procedures, technique modifications, help from an assistant)	Leave due to ERIs: 2 weeks
[17]	46%/-/- *as pain* 32%/-/- *as injury*	Neck (27% ^2^), lower back (18% ^2^), wrist (12% ^2^), hand (11% ^2^), mid-upper back (9% ^2^), finger (8% ^2^), shoulder (8% ^2^) *Indicated as pain location*	DQT (33% ^2^), cervical radiculopathy (26% ^2^), lumbar radiculopathy (20% ^2^), CTS (12% ^2^), ulnar nerve entrapment (6% ^2^), radial nerve entrapment (3% ^2^)	-	-	-	-
[18]	95% ^1,3^/-/-	Lower back (41% ^1^), leg (25% ^1^), hand (23% ^1^), thumb (22% ^1^), wrist (13% ^1^), neck (10% ^1^) *Indicated as pain location*	*Pain distributed as reported in “Location”*	-	Oral medication (10% ^1^), locally applied treatment (7% ^1^), no treatment (5% ^1^)	-	-
[19]	79% ^1^/-/-	Neck (47% ^1^), low back (42% ^1^), right shoulder (28% ^1^), left shoulder (27% ^1^)	-	17% ^1^	10% ^1^ sought medical care	-	-
[20]	75% ^1^/-/-	Thumb (63% ^1^), neck (59%), hand/finger (57% ^1^), lower back (53% ^1^), shoulder (47%), wrist (45%)	♀/♂ ^5^ Thumb pain (68% ^1^/61% ^1^), hand/finger pain (64% ^1^/53% ^1^), wrist pain (53% ^1^/41% ^1^), elbow pain (28% ^1^/34% ^1^), upper back pain (49% ^1^/36% ^1^), lower back pain (48% ^1^/55% ^1^), hand/arm numbness (39% ^1^/33% ^1^), DQT (26% ^1^/18% ^1^), CTS (25% ^1^/19% ^1^)	21% ^1^	Most frequently used treatment for ERI type: massage—upper back pain (38% ^1^), medication—lower back pain (35% ^1^), physiotherapy—shoulder pain (30% ^1^), splint—CTS (29% ^1^), rest—tendonitis (22% ^1^), steroid—tendonitis (19% ^1^), surgery—CTS (16% ^1^), acupuncture—lower back pain (3% ^1^) Surgery: 12% ^1^	-	A total of 20% ^1^ reported pregnancy during practice, with new ERIs (79% ^1^) or worsening of existing ones (70% ^1^), with 90% ^1^ with more than one ERI location.
[21]	53% ^1^/75% ^1^/46% ^1^	Neck (46% ^2^), shoulder (36% ^2^), thumb (36% ^2^), wrist (32% ^2^), lower back (30% ^2^), upper back (29% ^2^), elbow (18% ^2^), knee (6% ^2^), hip (3% ^2^), ankle (3% ^2^)	Muscle/tendon strain ^4^ (35%), tension neck syndrome ^4^ (20%), tendonitis ^4^ (18%), trigger finger/thumb ^4^ (12%), mechanical back syndrome ^4^ (9%), CTS ^4^ (8%), radial tunnel syndrome ^4^ (7%), rotator cuff tendonitis ^4^ (4%), ruptured/herniated disc ^4^ (4%), epicondylitis ^4^ (4%), ligament sprain ^4^ (3%), degenerative disc disease ^4^ (3%), DQT ^4^ (2%), digital neuritis ^4^ (2%), thoracic compression ^4^ (1%)	30%	No treatment ^4^ (48%), analgesics ^4^ (27%), NSAID ^4^ (26%), exercise ^4^ (19%), rest ^4^ (16%), physiotherapy ^4^ (14%), chiropractic ^4^ (4%), splint/braces ^4^ (3%), injections ^4^ (2%) Surgery ^4^: 2%	-	Leave due to ERIs: 1–2 days: 16% 3–7 days: 6% 7–15 days: 4% 15–30 days: 1% >30 days: 5%
[22]	84% ^1^/30% ^1^/-	Hand/wrist/finger (50%), back (38% ^1^), shoulder (30% ^1^), foot/plantar fasciitis (26% ^1^), elbow/forearm (19% ^1^)	*Pain distributed as reported in “Location”*	-	-	14% ^1^ limited or stopped the procedure	A total of 47% ^1^ had more than one ERI location, with an overall 2 ± 1 ^1^ ERI locations.
[23]	55% ^1^/60% ^1^/49% ^1^	Thumb (59% ^1^), hand/finger (57% ^1^), wrist (48% ^1^), lower back (44% ^1^), upper back (40% ^1^), neck (39% ^1^) *Additional location not reported.*	Hand/arm numbness (22% ^1^), CTS (10% ^1^), DQT (9% ^1^) *Pain distributed as reported in “Location”* ^5^	5% ^1^	Most frequently used treatment for ERI type: massage—neck pain (39% ^1^), rest—CTS (33% ^1^), splint—CTS (33% ^1^), medications—CTS (22% ^1^), physiotherapy—shoulder pain (14% ^1^), steroid—DQT (13% ^1^), surgery—CTS (11% ^1^), acupuncture—lower back pain (3% ^1^)	-	Five trainees reported pregnancy during their fellowship. A total of 80% reported that ERIs began or worsened (60%) during pregnancy, with 20% reporting additional time for procedures during pregnancy.
[24]	75%/-/- Endoscopists: 54% ^1^/-/- Nurses: 87% ^1^/-/-	Endoscopists ^5^: Neck/upper back ^3^ (23% ^1^), both shoulders ^3^ (15% ^1^), lower back ^3^ (15% ^1^), left thumb ^3^ (15% ^1^) Nurses ^5^: Lower back (53% ^1^), neck/upper back (40%), both shoulders (20%), left hand (13% ^1^), both hands (13% ^1^) *Indicated as pain location*	Endoscopists: Left hand numbness (8% ^1^) Nurse: CTS (13% ^1^), left-hand numbness (7% ^1^), both-hand numbness (7% ^1^) *Pain distributed as reported in “Location”*	21% ^1^	Medications (34% ^1^)	-	An ERI focus on pain and numbness. A total of 52% ^1^ could not be certain if this was work-related with 14% ^1^ saying that it was not.
[25]	77% ^1^/-/-	Neck (54% ^1^), back (50% ^1^), shoulder (39% ^1^), thumb (33% ^1^), arm (19% ^1^), finger (19% ^1^), elbow (18% ^1^), wrist (16% ^1^), knee (13% ^1^), hip (10% ^1^), other (4% ^1^)	-	10% ^1^	Analgesics (36% ^1^), physiotherapy (26% ^1^), other (5% ^1^) Surgery (3% ^1^)	(Adjustable bed and monitor, technique modification, increasing breaks, procedure reduction, orthopaedic aid, physical exercise)	-
[26]	85% ^1^/-/-	-	-	-	-	-	-
[27]	35% ^1^/44% ^1^/23% ^1^	Neck/upper back (44% ^1^), thumb (42% ^1^), hand/finger (38% ^1^), lower back (36% ^1^), wrist (18% ^1^), shoulder (18% ^1^), foot (10% ^1^), elbow (8% ^1^), knee (4% ^1^), knuckle (4% ^1^) *Indicated as pain location ^5^*	Hand/arm numbness (14% ^1^), tennis elbow (6% ^1^), CTS (4% ^1^), DQT (4% ^1^) *Pain distributed as reported in “Location”* ^5^	8% ^1^	24% received treatment ^6^	32% adjusted the practice ^6^	-
[28]	14% ^1^/13% ^1^/14% ^1^	Hand (79% ^1^), neck/shoulder (41% ^1^), back (38% ^1^), foot (15% ^1^), leg (14% ^1^)	*Pain distributed as reported in “Location”* ^5^	-	-	-	A total of 61% ^1^ who were pregnant during training reported having increased ergonomic difficulty during pregnancy.
[29]	50% ^1^/63% ^1^/41% ^1^	Hand/thumb/finger (40% ^1^), wrist (17% ^1^), shoulder (17% ^1^), neck (14% ^1^), lower back (14% ^1^), upper back (10% ^1^), elbow (8% ^1^), knee (5% ^1^), foot (4% ^1^), hip (3%), ankle (1% ^1^) *Indicated as pain location*	-	8% ^1^	-	-	A total of 73% ^1^ performed endoscopy during pregnancy, with 23% ^1^ also performing ERCP. (Modifications: procedure reduction; performing the endoscopy sitting.)
**Nasal endoscopy**							
[30]	72%/-/-	Back and neck (29%), neck (24%), back (19%) *Indicated as pain location*	-	-	Physiotherapy (83%), osteopathy (31%), chiropractic (17%)	-	-
[31]	62%/87% ^1^/58% ^1^	Neck (60% ^1^), back (56% ^1^), hand/wrist (19% ^1^), knee (13% ^1^), shoulder (13% ^1^), arm (11% ^1^), foot (8% ^1^), leg (3% ^1^), hip (3% ^1^)	Pain (84% ^1^), stiffness (60% ^1^), fatigue (48% ^1^), numbness (27% ^1^)	-	Stretching (24% ^1^), medication (6% ^1^), splint (2% ^1^), rest (2% ^1^) Surgery (29% ^1^)	(Procedure reduction, change of posture)	-
[32]	77%/-/-	Shoulder (63%), hand (54%), neck (46%), wrist (31%), lower back (29%), arm (21%), leg (19%)	Stiffness (65%), cramping (35%), tingling (25%)	-	23% sought medical care	(Technique modification, adjustable monitor, increasing breaks)	-
[33]	77% ^1^/-/-	Neck (60% ^1^), back (60% ^1^), shoulder (51% ^1^), finger (18% ^1^), wrist (12% ^1^), thumb (11% ^1^), knee (11% ^1^)	Pain (63% ^1^), stiffness (55% ^1^) (traumatic thoracic vertebral fracture, avascular necrosis of the humerus osteoarthritis, lateral epicondylitis)	8% ^1^	27% ^1^ sought medical care	-	-
[34]	71% ^1^/-/-	-	Musculoskeletal disorder ^5^ (47% ^1^)	23% ^1^	-	-	-
[35]	88% ^1^/-/-	Neck (41%), upper back (37%), low back (33%), wrist/hand (23%), shoulder (16%), elbow (10%) for outpatient Low back (41%), neck (33%), upper back (23%), wrist/hand (16%), elbow (15%), shoulder (15%) for OR	-	8% ^1^	-	(Adequate breaks, using back rest, skilled assistance, change in posture, customised workstation, time management)	A total of 81% for outpatient rooms and 64% ^1^ for ORs had pain in multiple sites.
[36]	84% ^1^/-/-	Neck (82% ^1^), lower back (56%), upper back (40% ^1^), shoulder (40% ^1^)	Neck stiffness ^5^ (72% ^1^), neck pain ^5^ (62% ^1^), lower back pain ^5^ (48% ^1^), lower back stiffness ^5^ (47% ^1^)	6% ^1^	-	(Procedure termination)	-
[37]	97%/-/-	Neck (64% ^1^), lower back (64% ^1^), upper back (58% ^1^) *Indicated as pain location*	Herniated disk, CTS, spondylolysis, brachial plexus compression, cervical spinal stenosis, lumbosacral radiculopathy with narrowing foramina, shoulder tendinopathy, bursitis, and epicondylitis	15%	Physiotherapy (29%), medication (29%), massage (20%), chiropractic (7%), osteopathy (5%) Surgery (7%)	-	Leave due to ERIs: 1–210 days
[38]	64% ^1^/-/-	Neck (82% ^2^), shoulder (52% ^2^), back (49% ^2^), hand (16% ^2^), leg (11% ^2^), arm (10% ^2^)	-	-	Physiotherapy (10% ^1^), exercise (20% ^1^), massage/chiropractic (20% ^2^) Surgery (7% ^1^)	4% ^1^ reduced the procedure due to ERIs	Injury rate specific for rhinolaryngoscopy
[39]	90% ^1^/93% ^2^/87% ^2^	Back (60% ^1^), neck (49% ^1^), shoulder (19% ^1^), hand (16% ^1^)	Pain (80% ^1^), stiffness (52% ^1^), fatigue (49% ^1^), numbness (41% ^1^)	-	61% ^1^ received treatment (pharmacotherapy, physiotherapy, osteopathy, chiropractic) Surgery (3% ^2^)	10% ^1^ made a modification (procedure reduction)	-
[40]	73% ^1^/-/-	Neck pain (6% ^1^ (sitting), 25% (standing)), back pain (19% ^1^ (standing)), limb (6% ^1^ (sitting) 6% ^1^ (standing))	-	-	-	-	-
[41]	91% ^1^/-/-	Shoulder (64% ^1^), neck (60%), lower back (58% ^1^), upper back (42% ^1^), ankle/foot (31% ^1^), knee (31% ^1^), hip/thigh (29%), elbow (9%)	-	-	-	-	-
[42]	35%/-/-	Back and neck (17%), neck (9%), back (9%) *Indicated as pain location*	-	-	-	-	-
**Bronchoscopy**							
[43]	51%/61%/49%	Neck (47%), lower back (46%), upper back (35%), left wrist (28%), right wrist (21%), right shoulder (19%), left shoulder (17%), left hand (15%), left finger (11%), left arm (9%), right finger (9%), right hand (7%), right arm (2%)	-	6%	Pain medication: 54% 17% sought medical care	(Modified workspace, procedure reduction, procedure termination)	Number of ERI location: 3 (2–4)
**Endourology**							
[44]	64% ^1^/-/-	Back (38% ^1^), neck (28% ^1^), hand (17% ^1^), hip/knee/ankle (14% ^1^)	Chronic intermittent backache, lumbar disc prolapse, lumbar spondylosis, pain, stiffness, cervical spondylosis, cervical disc prolapse, hand tremors, arthritis	-	-	-	Multiple injuries among 7%
[45]	33% ^1^/-/-	*Only hand or wrist problems are surveyed*	Numbness (13%), musculoskeletal pain (9%), joint pain (7% ^1^), lateral epicondylitis (4% ^1^), CTS (4% ^1^), ganglion cysts (3% ^1^), joint stiffness (3% ^1^), neuropathic pain (2% ^1^), hand arthritis (1% ^1^), DQT (1% ^1^), Dupuytren’s contracture (1% ^1^), trigger finger (1% ^1^)	-	Medications (21% ^1^), no treatment (15% ^1^), physiotherapy (5% ^1^) Surgery (1% ^1^) *For urologists*	-	-
[46]	62% ^1^/-/-	Neck (59% ^1,4^), back (57% ^1,4^), shoulder (51% ^1,4^), leg (28% ^1,4^), arm (26% ^1,4^), thumb (25% ^1,4^), hand (21% ^1,4^), wrist (21% ^1,4^)	-	8% ^1^	-	-	Leave due to ERIs: <1 week: 6% ^1^ 1–2 weeks: 3% ^1^ 2–3 weeks: 1% ^1^ >4 weeks: 0% ^1^
[47]	45% ^2^/39% ^2^/46% ^2^	Back pain (78% ^2^), neck pain (53% ^2^), back/neck (31% ^2^)	-	-	-	-	-
[48]	69% ^2^/33% ^2^/70% ^2^	Neck (64%), back (57%), shoulder (48%), hand (40%), elbow (18%)	-	-	Use of ice, heat and stretching (53%), NSAID (20%), muscle relaxants (11%)	-	-

^1^ Value rounded for conciseness. ^2^ Calculated by the reviewers based on the published data of the article. ^3^ Value specific for endoscopists^4^ Data retrieved from graph. ^5^ Additional data reported in the original article. ^6^ Unspecified measure.
